# The effect of L-Arg supplementation on L-Arg/NO metabolic and AMPK/ACC-1 signalling pathways in adipose cells (3T3 L1)

**DOI:** 10.1007/s00726-025-03467-0

**Published:** 2025-08-05

**Authors:** Saranya Prashath

**Affiliations:** Centre for Genomic and Child Health, Queen Mary University of London, 4 Newark St, London, UK

**Keywords:** 3T3 L1, L-Arg, Nitric oxide synthase, Nitric oxide, AMPK, ACC-1

## Abstract

L-arginine (L-Arg) is metabolised in the cell to generate nitric oxide (NO) and citrulline via nitric oxide synthase (NOS). NO is an important cellular signalling molecule that regulates lipid and glucose metabolism. The biological availability of NO is affected by the NOS inhibitor; N^G^-nitro-L-Arg methyl ester (L-NAME) and the external NO donor; S-nitroso-N-acetyl-D, L-penicillamine (SNAP). Mouse adipocyte 3T3 L1 cells were cultured with 0, 400 and 800 µM L-Arg or control complete DMEM media. The impact of L-NAME (4 mM), and SNAP (100 µM) was also analysed. The cell fitness was similar and the mRNA levels of AMPK was increased and ACC-1 was decreased, whilst the activation of AMPK and ACC-1 was decreased upon the addition of exogenous L-Arg. Transcript and protein levels of AMPK and ACC-1 were regulated by addition of L-NAME and SNAP, however the impact of these targets was related to the concentration of L-Arg added to the cells and the culture time point of analysis. NO in the form of NO_2_^−^ in cell culture supernatant was elevated in 400 and 800 µM L-Arg cultures. L-NAME significantly inhibited NO production from adipose cells in a time-dependent manner and subsequently impacted AMPK and ACC expression. Associated with these changes were changed in the concentration of L-Arg, L-Cit and L-Orn in the culture media. Collectively, these results show that excess L-Arg is sensed by the cell which then regulates AMPK and ACC-1 expression in response. The findings could have implications in modulation of signalling pathways for treating obesity and obesity induced diabetic mellitus.

**Supplementary Information:**

The online version contains supplementary material available at 10.1007/s00726-025-03467-0.

## Introduction

The 20 amino acids are the key building blocks of proteins and peptides, but also play a role in other metabolic pathways in cells (Wu 2014). The study here focuses on eukaryotic cells, specifically mammalian cells (mouse adipose cells), and amino acid metabolism involving the amino acid L-Arg. L-Arg metabolism in the cell can give rise to urea production and ornithine via arginase activity or nitric oxide and L-citrulline production via nitric oxide synthase (NOS) (Rath et al. 2014). Three isoforms of nitric oxide synthase (NOS) are known; calcium-dependent and membrane associated endothelial NOS (eNOS or NOS3), calcium-dependent neuronal NOS (nNOS or NOS1) (Alderton et al. 2001), and calcium-independent cytosolic inducible NOS (iNOS or NOS2) (Alderton et al. 2001; Sessa 2004; Balligand et al. 2009). eNOS and nNOS are expressed constitutively at low amounts in a variety of cell types and tissues and are controlled by the level of Ca^2+^ binding with calmodulin (Wu and Morris 2008; Tengan et al. 2012). However, iNOS is not expressed, or very low level, in cells or tissues under ‘normal’ condition (Wu and Morris 2008) and instead its expression is induced under appropriate conditions (Alderton et al. 2001). Three isoforms of the NOS are expressed in various tissues, including insulin-sensitive tissues (liver, muscle and adipose tissue) that play an important role in whole-body homeostasis of energy substrates (Jobgen and Wu 2022).

Nitric oxide (NO), a free radical, is synthesised by 3 isoforms of NOS (Förstermann and Sessa 2012) and oxygen, from L-Arg and requires a number of co-factors including tetrahydrobiopterin [(6R)-5,6,7,8-tetrahydrobiopterin] (BH_4_), flavin adenine dinucleotide (FAD), flavin mononucleotide (FMN) and nicotinamide adenine dinucleotide phosphate (NADPH) (Tengan et al. 2012). BH_4_ is an essential cofactor of NOS, playing a crucial role in regulating endothelial NO synthesis (Luiking et al. 2011; Kohli et al. 2018). NO is modulated by a range of NOS inhibitors such as N^G^-nitro-L-Arg methylester (L-NAME), N^G^-monomethyl-L-Arg (L-NMMA) and N^G^-nitro-L-Arg (L-NNA) (Hon et al. 2015). Nitric oxide is an important intra- and inter-cellular (McAdam et al. 2012; Sansbury and Hill 2014) signalling molecule that regulates nutrient metabolism (Frühbeck and Gómez-Ambrosi 2001). Physiological levels of NO (25 to 35 μmol) (Pahlavani et al. 2017) stimulate glucose uptake and oxidation as well as fatty acid oxidation in insulin-sensitive tissues, inhibit the synthesis of glucose, glycogen and fat in target tissues (e.g. liver and adipose) and enhance lipolysis in adipocytes (Jobgen et al.^2006^). The production of NO occurs in nearly all cells and tissues of mammals, particularly adipocytes, endothelial cells, cardiac cells, neurons, hepatocytes, myotubes and phagocytic cell (Lee et al. 2003; Wu et al. 2015). Oxidative metabolite products of NO are nitrites and nitrates whose formation swiftly deactivates NO and which are diffused into circulation. As such, the previous existence of NO in circulation can be measured indirectly by determining the concentrations of nitrite and nitrate present and these are frequently used as indicators of the NO concentration (Hon et al. 2015).

Adipogenesis, the intake of glucose by the stimulation of insulin and degradation of lipids in adipose tissue is regulated by NO. If NOS activity is reduced, glucose uptake is severely diminished (Roy et al. 1998). This has also been shown in 3T3-L1 adipocyte cell lines, used as a model to investigate adipocyte-NO responses, nitric oxide induces glucose uptake via insulin-independent GLUT4 translocation (Tanaka et al. 2003). L-Arg supplementation was also shown to reduce adiposity and improve insulin sensitivity in animal models of obesity as well as in patients with diabetes and obesity (Forzano et al. 2023). There are many physiological functions associated with L-Arg supplementation in mammals, but we do not yet know its underlying mechanisms (Wu et al. 2000; Kong et al. 2012). Further to this, new approaches in managing and preventing diabetes in obese individuals must be studied and investigated based on less side effects and cost effective. In this study, we aimed to investigate the direct effect of excess L-Arg supplement in insulin sensitive cells (mouse adipose cells) by modulating the expression of key target genes and proteins which are involved in the metabolism of energy substrates in an NO dependent manner. Specifically, we focused that excess L-Arg modulates energy metabolism by enhancing NO production, by activating the major energy sensing and downstream genes AMPK and ACC-1, via the AMPK-ACC-1/NOS/NO pathway.

## Materials and methods

### Establishment of cell culture models for investigating cell fitness and AMPK and ACC-1 cell signalling upon addition of exogenous L-Arg

#### Preparation of stock solutions of L-Arg and growth medium with varying L-Arg concentrations

Commercially available DMEM media for Stable Isotope Labelling using Amino Acids (SILAC DMEM, Thermo Scientific, United States), which is deficient in both L-lysine and L-Arg, was used to generate growth medium with varying L-Arg concentrations. Foetal bovine serum (FBS, Sigma, United States), was added to a final concentration of 10% (v/v). L-lysine-HCl (Sigma, United States) was also added to give the same concentration of L-lysine (146 mg/L) as in the complete DMEM basal media formula described in Thermo Fisher Scientific company (DMEM, Cat. No: 41966, United States). This media was termed L-Arg deficient media, containing no exogenously added L-Arg (although FBS could contain unspecified concentrations of L-Arg, the amount of L-Arg in the media was determined by HPLC after FBS addition as undetectable and hence this media was considered L-Arg deficient (0 µM)). L-Arg (Sigma, United States, 0.125 g) was added to the SILAC L-Arg deficient DMEM media (10 mL) to obtain an L-Arg stock solution (71.75 mM). This was then diluted as required with the L-Lys and FBS containing SILAC media to generate different L-Arg concentration containing mediums.

#### Cell culture

3T3 L1 cell lines were used as model systems, which were a generous gift from Dr Arcidiacono Biagio, University of Catanzaro ‘Magna Graecia’, Italy. The 3T3 L1 cell line is a mouse adipocyte (mouse embryonic fibroblast) insulin-sensitive cell (Li et al. 2019; Søndergaard and Jensen 2016). 3T3 L1 cells were seeded into 6-well tissue culture plates at 2 × 10^5^ viable cells/well in 2 mL of complete DMEM media and then incubated in a static incubator for 24 h at 37 °C, 5% CO_2_. After 24 h, the media was replaced with media of different concentrations of L-Arg (0, 400 and 800 µM) or the control complete DMEM with 10% (v/v) FBS (2 mL/ well). Complete DMEM is meant to contain 398.10 µM of L-Arg hydrochloride according to the media formulation from the company (Thermo Fisher, United States), however this was measured as only 250 µM using the HPLC method used (studied). Thus the 400 and 800 µM culture conditions represent a 1.6 and 3.2-fold increase in exogenous L-Arg concentration compared to the complete DMEM controls. Cells were then harvested at time points; 24, 48, 72 and 120 h to measure culture viability (%) and the viable number of cells/mL (× 10^6^) using a Vi-CELL cell viability analyzer (Beckman Coulter, Life sciences, United States). The cells and cell culture supernatant were also harvested 24 and 72 h post addition of L-Arg media of different concentrations to analyse mRNA and protein expression and metabolite of L-Arg in cell culture supernatant. The 3T3 L1 cells and cell culture supernatant were also harvested at T = 0; untreated samples (24 h after incubation of the cells and collected before L-Arg addition).

In some experiments, after 24 h, the media was replaced with media with different concentrations of L-Arg (0, 400 and 800 µM) or the control complete DMEM with 10% (v/v) FBS (2 mL/ well) with addition of L-NAME (a NOS inhibitor, 4 mM) and cells and cell culture supernatant then harvested at two time points; 24 and 72 h post addition. In another set of experiments, after 24 h the media was replaced with media of different concentrations of L-Arg (0, 400 and 800 µM) or the control complete DMEM with 10% (v/v) FBS (2 mL/ well) with addition of SNAP (a NO donor, 100 µM) and cells and cell culture supernatant harvested 6 and 24 h post addition. Subsequently samples were differently lysed for mRNA and protein work. The cell culture media (2 mL) from addition experiments was also collected from the cell cultures and frozen at -20 °C.

#### Quantitative real-time PCR (qRT-PCR) for transcript mRNA analysis

3T3 L1 cells were lysed by lysis using RLT (RNA lysis buffer, 350 µL, Qiagen, Germany). Collected RNA lysates at T = 0, 24 and 72 h or 6 and 24 h were homogenised using a QIAshredder kit (Qiagen, Germany). Extraction of total RNA was then achieved using the commercially available RNeasy Mini Kit (Qiagen, Germany) following the protocol of the manufacturer. Extracted total RNA was treated for contaminating DNAase using RQ1 RNase-Free DNase kit (Promega, USA). Primers (forward and reverse, described in Table S1 in Supplementary Information SI) were designed to target genes of interest. qRT-PCR amplification of target sequences and the housekeeping control (β-actin) was undertaken using the commercially available iTaqTM Universal SYBR Green One-Step Kit (Bio-Rad, United States). A DNA engine opticon 2 system for real-time PCR detection thermocycler (Bio-Rad, USA) was used for amplification and for producing melting curve profiles of the amplicons.

#### Protein and post-translational modification (phosphorylation) analysis using western blotting

Samples for protein analysis were lysed by lysis buffer (200 µL/well). Bradford assay was used to determine protein concentration in samples. Samples were prepared with × 5 sample/loading buffer (reducing buffer). The ratios between reducing sample buffer (× 5): diluted sample was 1:5. The required volume of protein extract was diluted with water to obtain × 1 final sample buffer concentration with 10 and 20 µg of protein/25 µL total sample volume/lane. The proteins were separated using 10% sodium dodecyl sulfate–polyacrylamide gel electrophoresis. The protein is then transferred to nitrocellulose blotting membranes was performed using wet transfer conditions. The proteins were incubated with appropriate primary antibody (detailed in Table S2 in Supplementary Information SI) overnight at 4 °C. Then blot was probed with an appropriate secondary antibody conjugated to Horseradish peroxidase at room temperature for 1 h for development using an Optimax 2010 film processor (PROTEC GmbH & Co, Germany). Densitometry of bands on Western blots was undertaken using the open software package ImageJ (National Institutes for Health (NIH), USA).

#### Nitric oxide /nitrite measurement by Griess assay

Frozen cell culture media (2 mL), collected from L-Arg and/or L-NAME, and SNAP additions to 3T3 L1 cells, were vortexed and then centrifuged for 10 min at 1500 rpm and the supernatant without cell pellet was collected. Cell culture supernatant (50 µL) was used in the assay. Nitrite accumulation in cell culture supernatants after L-Arg supplementation was determined by Griess assay (Griess 1879 and Tsikas 2007) using sodium nitrite as a standard.

#### Analysis of L-arginine, L-ornithine and L-citrulline by HPLC

The collected serum samples were vortexed and centrifuged at 1500 rpm for 10 min to obtain the cell free culture supernatant for HPLC analysis. Samples were deproteinised using a modified protocol of that described by (Yang et al. 2014). To remove FBS and cellular proteins from the supernatant of the cell culture media, 100% (v/v) ice-cold ethanol (400 µL) was added to the cell culture supernatant (100 µL) followed by vortexing vigorously for 15 min at room temperature.

Amino acids were derivatized at room temperature using a pre-column derivatisation method. O-phthaldialdehyde (OPA) reagent complete solution consisting of OPA (Sigma, USA,1 mg/mL), was used as a pre-column derivatization agent, specially formulated for primary amines and amino acids at alkaline pH. Mobile phase A (0.1 M sodium acetate, pH 7.2) was prepared as described by (Wu and Meininger 2008). The mobile phase B was 100% (v/v) methanol. A standard amino acid mixture (6 mM), consisting of the essential 20 amino acids, was a gift from Dr Andrew Lawrence, School of Biosciences, University of Kent, and was prepared as outlined previously (Moore et al. 2018). Additionally, L-citrulline (Sigma, USA) and L-ornithine monohydrochloride (Sigma, USA) (0.6 mM of each) were added to the standard amino acid mixture from respective stock solution of L-citrulline and L-ornithine (6 mM of each) to form an extended amino acid standard solution.

To a dark glass vial (1.5 mL), an extended amino acid standard mixture (0.6 mM each) or test cell culture sample solution (100 µL) was added. An Agilent 1100 HPLC (Agilent Technologies, Germany) equipped with a diode-array detector (DAD) was used. Samples were injected (25 μL) onto an ACE HPLC column; RP-C18, dimensions 125 × 4.6 mm, 5 μm (Avantor, United States). The autosampler was automated to mix 25 µL of a standard or sample solution with 25 µL of the OPA reagent solution and allowed for incubation for 1 min in a reaction loop. Amino acids were separated with linear gradient (Table S3 in Supplementary Information SI) with a total running time of 49 min; flow rate, 1.1 mL/min. The detector, Diode Array Detector (DAD), was programmed to switch to 338 nm, 10 nm bandwidth, and reference wavelength 390 nm, 20 nm bandwidth. The molar absorptivity of each derivatized amino acid was detected at 338 nm (λmax). Standard curve of L-Arg, L-citrulline and L-ornithine were generated using the stock solutions of L-Arg, L-Cit and L-Orn. Identification of particular amino acid signals was based on the comparison between the retention time of the extended amino acid standard mixture and the amino acids of interest from analysis of samples. Quantitation was based on the standard curve method using a linear curve fitted by linear regression analysis. The unknown concentration of each amino acid in experimental samples was calculated using the equation of the line fitted for the standard curves of amino acids.

### Statistical analysis

Statistical analysis was undertaken using GraphPad Prism 9.4.1 software for Windows (GraphPad Software, San Diego California USA) and Microsoft Excel. Samples were analysed in triplicate biological replicates. Data interpreted of means and standard deviation were analysed using two-way ANOVA. The Tukey’s multiple comparison method was used to determine differences among the means of the treatment groups (0, 400 and 800 µM L-Arg) with the control complete DMEM media addition at the time points 24 and 72 h. To compare between multiple treatments groups, the Bonferroni’s multiple comparison method was used to determine differences among the means of the treatment groups (0, 400 and 800 µM L-Arg) and the control complete DMEM media addition with the addition of either L-NAME or SNAP in 3T3 L1 cells across the time points either 24 and 72 h or 6 and 24 h. Probability values ≤ 0.05 were considered to indicate statistical significance.

## Results

### Impact of L-Arg addition on culture viability and viable cell numbers in 3T3 L1 cells

**Fig. 1:**
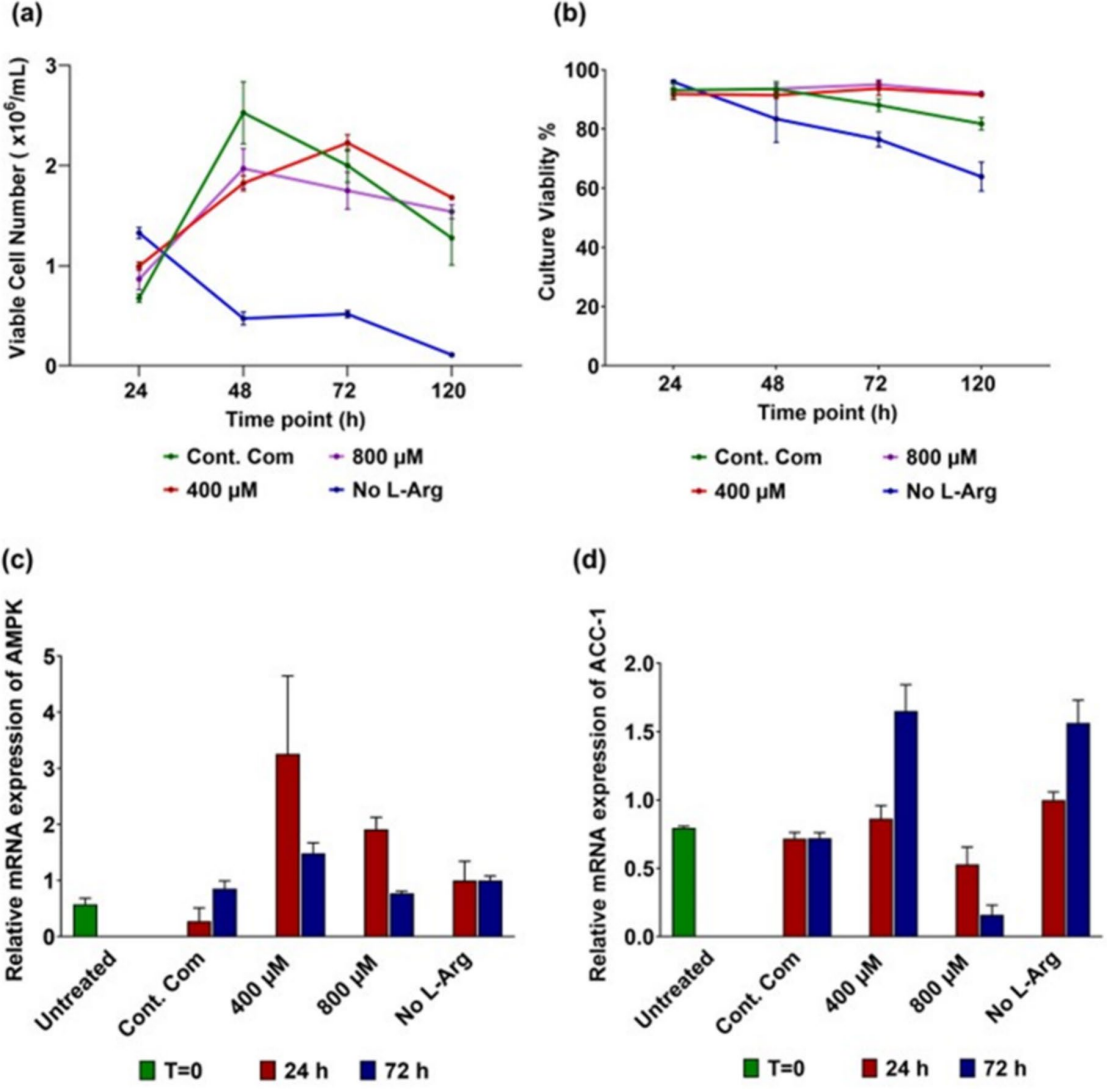
Cell growth and culture viability profiles and relative mRNA expression in 3T3 L1 cells. Cell growth/viable cell number (**a**) and culture viability (**b**) profiles of 3T3 L1 cells with different concentrations of L-Arg (0, 400 and 800 µM) and the control complete DMEM media at 24, 48, 72 and 120 h. Relative mRNA expression of AMPK (**c**) and ACC-1 (**d**) cultured in different concentrations of L-Arg in 3T3 L1 cells at 24 and 72 h. Data points represent the mean ± SD of each culture sample. Error bars represent the standard deviation from the mean (n = 3)

The viable number of cells (Fig. 1a) shows that there was an increase in 3T3 L1 cell numbers from 24 to 48 h except in the no L-Arg supplemented cell samples. However, thereafter there was a decline in 3T3 L1 cell numbers from 48 to 120 h in all samples and conditions except the 400 µM L-Arg supplemented cell samples. Overall, in comparison to the control, an absence of exogenous L-Arg decreased the viability of 3T3 L1 cultures. Regardless of the growth profiles (Fig. 1a), the effect of L-Arg (0, 400 and 800 µM) compared to the control complete DMEM media was significant (P < 0.0001) as determined by two-way ANOVA analysis of the means values of the viable cell numbers at the different time points followed by a Tukey multiple comparison test.

Culture viability (Fig. 1b) of the samples and conditions was generally maintained between 80–95% across the 120 h except no-L-Arg SILAC DMEM (0 µM) cultures. It is interesting to note that the culture viability of 400 and 800 µM L-Arg cultures followed the same pattern. In comparison to other culture conditions, cultures with the lowest concentration of L-Arg (0 µM) decreased in culture viability rapidly. The presence of excess L-Arg (400 and 800 µM) compared to the control complete DMEM media was significant (P < 0.0001) as determined by two-way ANOVA analysis of the means values of the culture viability at the different time points followed by a Tukey multiple comparison test.

### qRT-PCR analysis of AMPK and ACC-1 genes in response to L-Arg supplementation to 3T3 L1 cells

Relative gene expression analysis (∆∆Ct) was used to quantify target gene mRNA expression. The target gene expression was normalized to β-actin to obtain the ∆Ct and then the relative difference (∆Ct) between target and β-actin was normalized to the ∆Ct value of the control no L-Arg added sample at the 24 h culture time point to obtain the ∆∆Ct value. The relative expression of each gene was determined and denoted as RE. The target transcripts AMPK and ACC-1, which are hypothetically modulated by changes in L-Arg through signalling pathways, were investigated. From the qRT-PCR data (Fig. 1c), AMPK gene expression was increased (P < 0.0001) in 400 µM L-Arg addition cultures at 24 (RE 3.26) and 72 h (RE 1.49) when compared to the control complete DMEM media cultures. AMPK mRNA expression also increased (P < 0.0001) in 800 µM L-Arg cultures (RE 1.91) at 24 h in comparison to the control (RE 0.28), however, the AMPK expression in both 800 µM and the control samples was more or less the same at 72 h. AMPK mRNA expression was more-or-less unchanged in no L-Arg added samples at 24 and 72 h time points. The mRNA levels of the key lipogenic enzyme, ACC-1, was slightly increased (P < 0.0001) in cultures with arginine at 400 µM (RE 0.86) compared to the control (RE 0.72) and increased (P < 0.0001) at 72 h (RE 1.65) compared to the control complete DMEM media cultures (RE 0.72) (Fig. 1d). Noticeably, there was no difference in ACC-1 mRNA expression within the time points 24 (RE 0.72) and 72 h (RE 0.72) in the control complete DMEM. ACC-1 gene expression was decreased (P < 0.0001) in 800 µM L-Arg cultures at 24 (RE 0.53) and 72 h (RE 0.16) in comparison to the control (at 24 and 72 h; RE 0.72) and no L-Arg added samples (at 24; RE 1 and 72 h; RE 1.56).

### qRT-PCR analysis of AMPK and ACC-1 genes upon L-NAME (4 mM), and SNAP (100 µM) addition to 3T3 L1 cells

**Fig. 2:**
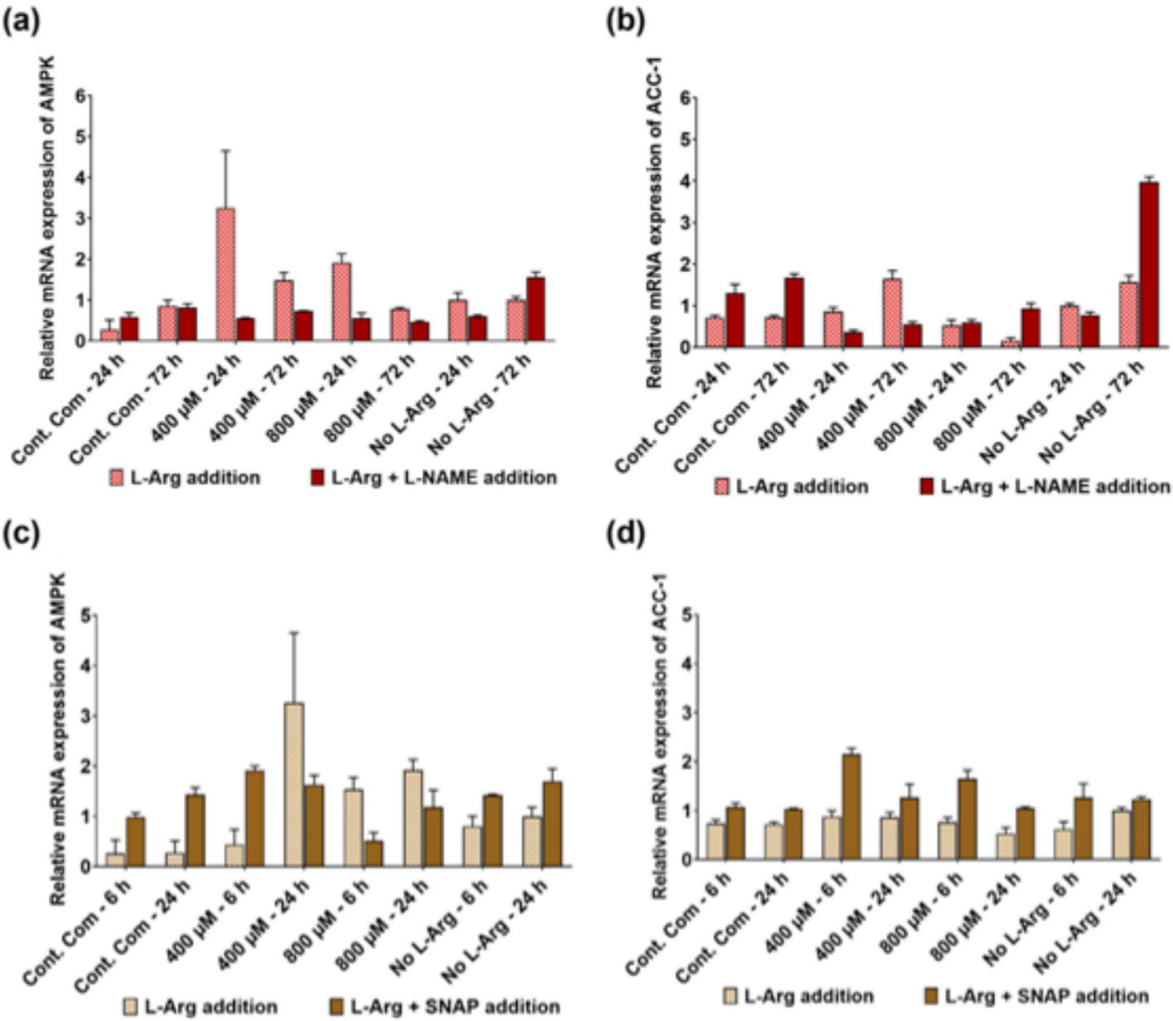
Regulation of relative mRNA expression of AMPK and ACC-1 in 3T3 L1 cells. Relative mRNA transcript expression (∆∆Ct) of AMPK (**a**) and ACC-1 (**b**) in the 3T3 L1 cells with nitric oxide synthase inhibitor; L-NAME (4 mM) 24 and 72 h after addition, and relative mRNA transcript expression of AMPK (**c**) and ACC-1 (**d**) in the 3T3 L1 cells with nitric oxide donor; SNAP (100 µM) 6 and 24 h after addition. All these analyses were undertaken in 3T3 L1 cells cultured in medium of different L-Arg concentrations (0, 400 and 800 µM) and control complete DMEM media. Data points represent the mean ± SD of each sample. Error bars represent the standard deviation from the mean (n = 3)

When analysis the impact of L-NAME addition on AMPK mRNA levels in 3T3 L1 cells in different exogenous L-Arg concentrations, L-NAME treated samples (Fig. 2a) cultured with excess exogenous L-Arg (400 and 800 µM) showed decreased (P < 0.0001) AMPK gene expression for 24 and 72 h in comparison to the control samples. The mRNA levels of ACC-1 (Fig. 2b) were increased (P < 0.0001) in L-Arg at highest concentration (800 µM) with L-NAME (24 h; 1.13-fold and 72 h; 5.81-fold) and the control complete DMEM with L-NAME (24 h; 1.81-fold and 72 h; 2.32-fold).

There was no significant difference (P = 0.1835) in the expression of AMPK mRNA between the samples cultured with or without SNAP Fig. 2c. Overall comparison between the samples cultured with SNAP and without SNAP (Fig. 2d) revealed that there was an increase (P < 0.0001) in ACC-1 mRNA in the samples treated with SNAP.

### AMPK and ACC-1 protein and their post-translational phosphorylation expression in response to L-Arg supplementation to 3T3 L1 cells

**Fig. 3:**
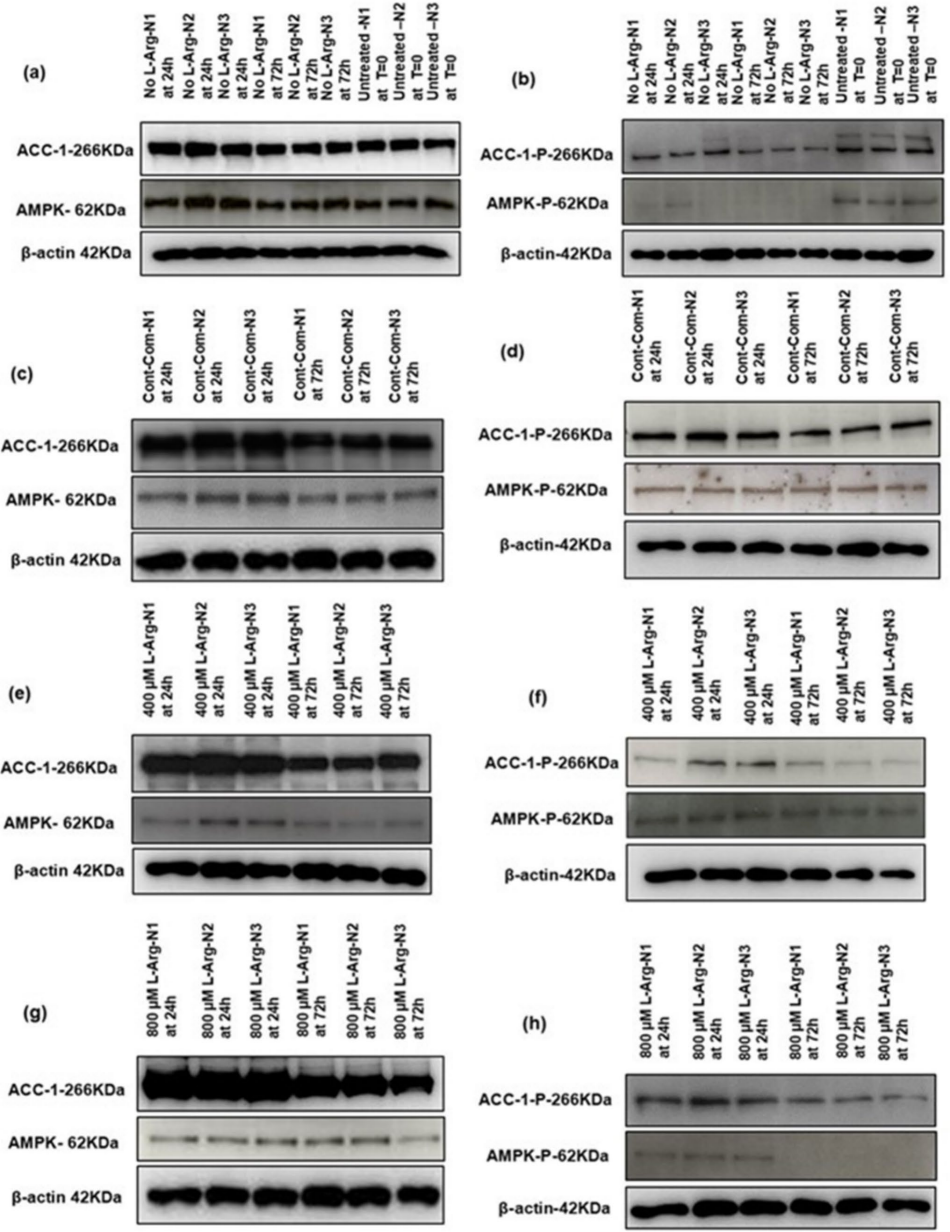
Western blot comparison for target proteins and phosphorylated proteins expression in 3T3 L1 cells. Western blot comparison of the expression of AMPK and ACC-1 proteins (**a**, **c**, **e** and **g**) involved in L-Arg/NO metabolic pathway signalling, and the amount of phospho-protein (**b**, **d**, **f** and **h**) of these targets in 3T3 L1 cells cultured in no L-Arg SILAC DMEM media, control complete DMEM media and 400 and 800 µM L-Arg in L-Arg free SILAC DMEM media for 24 and 72 h and in one of the control, untreated culture samples at T = 0. β-actin is used as a loading control. The same amount of protein (10 µg) from different treatment groups was loaded from biological triplicate cultures into 10% SDS–polyacrylamide gels for the separation of target proteins

The gels were used to allow a comparison within a concentration with changing culture time (Figs. 3a–h). Target protein expression was normalized to a reference protein; β-actin protein. All data was then normalized to the value of no L-Arg added samples (0 µM L-Arg) at 24 h culture time point, denoted as RE in the following sections.

**Fig. 4:**
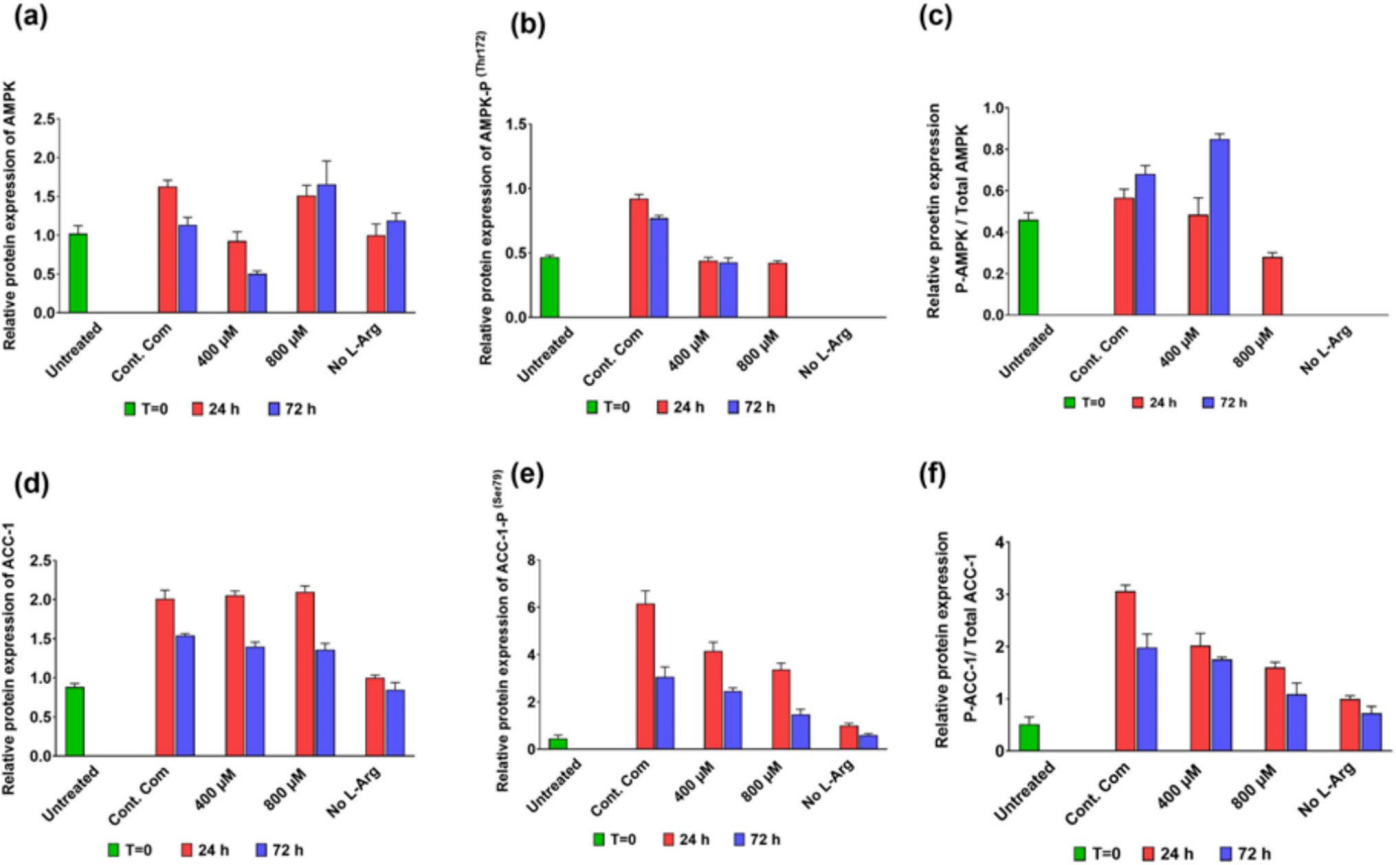
Relative total and phosphorylated protein expressions of AMPK and ACC-1 in 3T3 L1 cells. Relative protein amounts for total AMPKα (**a**) and ACC-1 (**b**), phosphorylated AMPKα at Thr172 (AMPKα-P) (**c**) and ACC-1 at Ser79 (AMPKα-P) (**d**) and the ratio of AMPKα-P to total AMPKα in 3T3 L1 cells (**e**) and ACC-1-P to total ACC-1 in 3T3 L1 cells (**f**). Cells were cultured for 24 or 72 h in customized media containing 0, 400 and 800 µM L-Arg. Controls were addition of complete DMEM or untreated cultures at T = 0. Bands in the blots were quantified using ImageJ software and the data are normalised to β-actin and then expressed as relative to the no L-Arg SILAC DMEM (0 µM L-Arg) control at 24 h. Data points represent the mean ± SD. Error bars represent the standard deviation from the mean (n = 3)

The AMPK protein expression (Fig. 4a) showed increased and peaked expression (P < 0.0001) when cultured in 800 µM (1.5-fold) L-Arg after 72 h compared to the control complete media samples at 72 h. AMPK protein expression was decreased (P < 0.0001) when cultured in 400 µM at 24 (RE 0.93) and 72 h (RE 0.50) compared to the control complete DMEM media additions at 24 (RE 1.63) and 72 h (RE 1.13). AMPK protein expression in no L-Arg media samples at 72 h (RE 1.19) was more-or-less the same as the control at 72 h. When investigating the phosphorylation of AMPK (Fig. 4b), in 400 µM L-Arg cultured samples phosphorylation was very similar (P < 0.0001) at 24 (RE 0.52) and 72 h (RE 0.53) and more or less the same when compared to untreated samples at T = 0 (RE 0.58). There was no (P < 0.0001) detectable phosphorylated AMPK protein in no L-Arg media samples. Increasing L-Arg concentrations to 800 μM resulted in decreased (P < 0.0001) phosphorylated AMPK levels at 24 (RE 0.52) and 72 h (RE 0) in comparison to the control at 24 (RE 1.14) and 72 h (RE 0.95). When comparing phosphorylated AMPK protein to total AMPK protein (Fig. 4c), the ratio was increased in 400 µM L-Arg (1.25-fold) cultures at 72 h in comparison to the control at the same time point.

The expression of ACC-1 protein (Fig. 4d), regulator of fatty acid metabolism, decreased (P < 0.0001) with the time in cells cultured in L-Arg at 400 and 800 μM and in the control. Relative ACC-1 protein expression in the control, 400 and 800 µM L-Arg cultures (P < 0.0001) at 24 h was 2.0, 2.0 and 2.10 respectively, whereas the relative ACC-1 protein expression for these samples at 72 h was decreased (P < 0.0001) as 1.54, 1.4 and 1.36. After 72 h of culture time total ACC-1 amounts in the untreated sample at T = 0 (RE-0.88) and no L-Arg added samples (RE 0.85) had similar and the lowest relative expression of ACC-1 among all samples. When phosphorylation of ACC-1 (Fig. 4e) was investigated, this was almost halved from its level at 24 h to 72 h. The relative expression of phosphorylated ACC-1 in the control, 400 and 800 µM L-Arg cultures at 24 h were 6.16, 4.15 and 3.36 and at 72 h were 3.06, 2.45 and 1.47 respectively. Among all samples, expression of phosphorylated ACC-1 was higher in the control (RE 6.16) at 24 h and the amounts of phosphorylated ACC-1 was increased (P < 0.0001) (twofold) in comparison to the 800 µM L-Arg added samples at 24 and 72 h time points. Phosphorylated ACC-1 amounts in the untreated sample at T = 0 (RE 0.46) and no L-Arg cultures (RE 0.60) were the lowest relative expression of phosphorylated ACC-1 among all samples. The ratio of phosphorylated ACC-1 to total ACC-1 was increased (P < 0.0001) in the control at 24 (1.9-fold) and 72 h (1.82-fold) compared to the additions of 800 µM L-Arg at the same time points (Fig. 4f).

### AMPK and ACC-1 protein and their post-translational phosphorylation expression in response to L-Arg supplementation to 3T3 L1 cells with L-NAME (4 mM) and SNAP (100 µM)

**Fig. 5:**
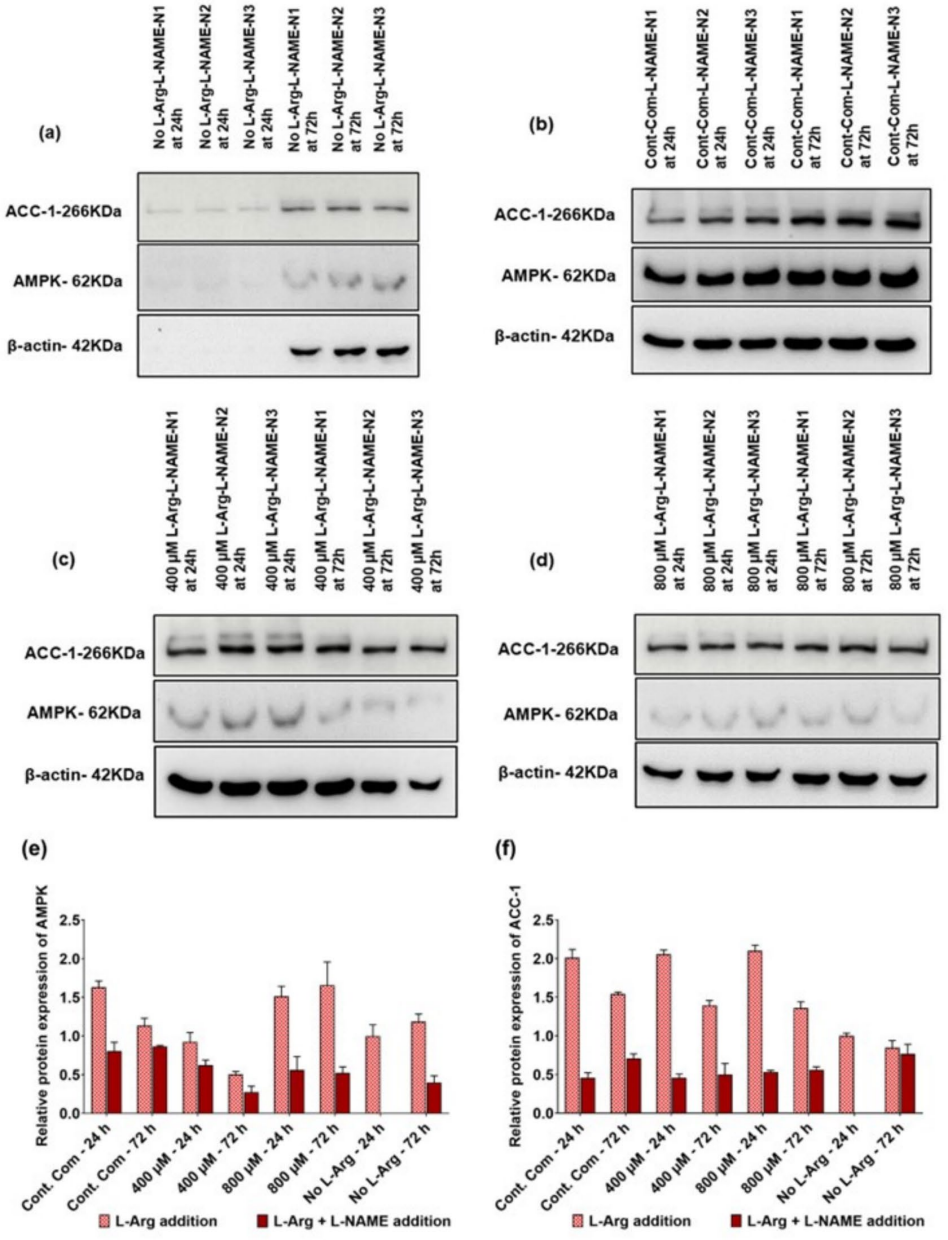
Western blot and relative AMPK and ACC-1 protein expressions in 3T3 L1 cells with L-NAME. Western blot comparison of the expression of key protein; AMPK and lipogenic ACC-1 protein levels investigated in L-Arg/NO metabolic pathway signalling in 3T3 L1 cells cultured in customized media containing **a** no L-Arg SILAC DMEM and L-NAME (4 mM), **b** complete DMEM and L-NAME (4 mM), **c** 400 µM L-Arg and L-NAME (4 mM), and **d** 800 µM L-Arg and L-NAME (4 mM) for 24 and 72 h. Relative protein expression of AMPK (**e**) and ACC-1 (**f**) involved in L-Arg/NO metabolic pathway signalling in 3T3 L1 cells cultured in different concentrations of L-Arg and the control with or without L-NAME for 24 and 72 h conditions investigated. β-actin is used as a loading control. The same amount of protein (10 µg) from different treatment groups was loaded from biological triplicate cultures into 10% SDS–polyacrylamide gels for the separation of target proteins. Data points represent the mean ± SD. Error bars represent the standard deviation from the mean (n = 3)

**Fig. 6:**
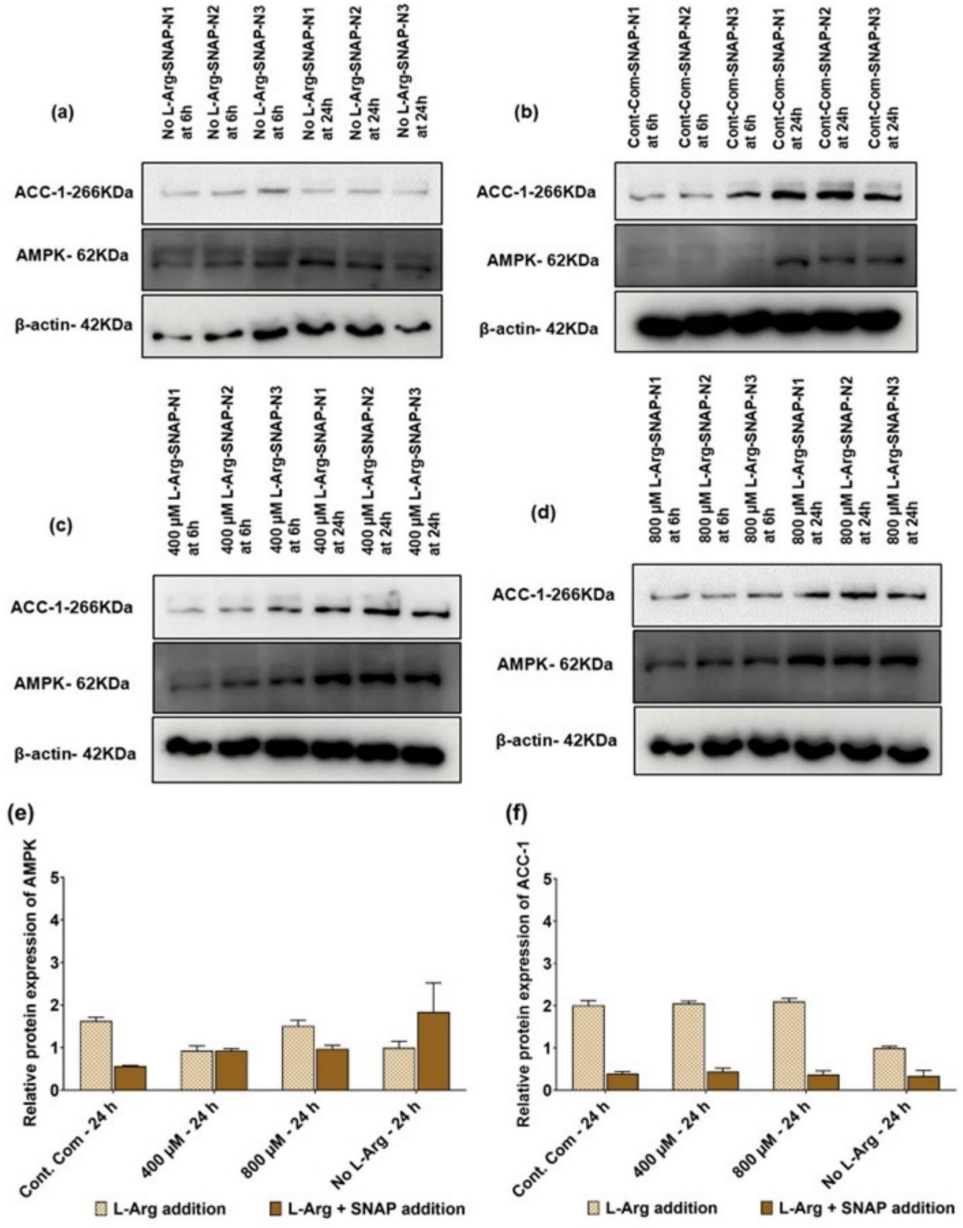
Western blot and relative AMPK and ACC-1 protein expressions in 3T3 L1 cells with SNAP (100 µM). Western blot comparison of the expression of key protein; AMPK and lipogenic ACC-1 protein levels investigated in L-Arg/NO metabolic pathway signalling in 3T3 L1 cells cultured in customized media containing **a** no L-Arg SILAC DMEM and SNAP (100 µM) **b** complete DMEM and SNAP (100 µM), **c** 400 µM L-Arg and SNAP (100 µM), and **d** 800 µM L-Arg and SNAP (100 µM) for 6 and 24 h. Relative protein expression of AMPK (**e**) and ACC-1 (**f**) involved in L-Arg/NO metabolic pathway signalling in 3T3 L1 cells cultured in different concentrations of L-Arg and the control with or without SNAP for 24 h conditions investigated. β-actin is used as a loading control. The same amount of protein (10 µg) from different treatment groups was loaded from biological triplicate cultures into 10% SDS–polyacrylamide gels for the separation of target proteins

In 3T3 L1 cells, protein expression (AMPK and ACC-1) was also investigated in L-NAME (Fig. 5a–d), and SNAP (Fig. 6a–d) additions to 3T3 L1 cells.

The relative protein expression of AMPK upon L-NAME addition to different exogenous L-Arg concentrations is presented in Fig. 5e. Overall, the addition of L-NAME reduced (P < 0.0001) the AMPK protein expression. The relative protein expression of ACC-1 upon L-NAME addition is presented in Fig. 5f. Like AMPK protein expression, overall, L-NAME addition to the 3T3 L1 cells decreased (P < 0.0001) the ACC-1 protein expression in all the cell samples treated with L-NAME at 24 (the control + L-NAME; 0.23-fold, 400 µM + L-NAME; 0.22-fold, 800 µM + L-NAME; 0.25-fold and 0 µM + L-NAME; 0) and 72 h (the control + L-NAME; 0.46-fold, 400 µM + L-NAME; 0.36-fold, 800 µM + L-NAME; 0.41-fold and 0 µM + L-NAME; 0.91-fold) compared to the samples without L-NAME.

When analysis the changes in AMPK and ACC-1 protein expression upon SNAP addition to the 3T3 L1 cells cultured in different concentrations of L-Arg and the control complete DMEM, AMPK protein expression did not change significantly (P = 0.0908) between samples cultured in excess L-Arg (400 and 800 µM) with and without SNAP addition Fig. 6e. There was a large decrease (P < 0.0001) in ACC-1 protein expression (Fig. 6f) in the samples cultured in different concentrations of L-Arg (0, 400 and 800 µM) and the control complete DMEM followed by SNAP addition at 24 h (the control + SNAP; 0.2-fold, 400 µM + SNAP; 0.21-fold, 800 µM + SNAP; 0.18-fold and 0 µM + SNAP; 0.33-fold) compared to the cell samples cultured without SNAP. Across the culture time the relative expression of ACC-1 protein was increased (P < 0.0001) in L-Arg with SNAP (400; 2.1-fold and 800 µM; 1.85-fold) whereas, decreased (P < 0.0001) in the control (0.3-fold) and no L-Arg (0.63-fold) cell samples treated with SNAP.

### Determination of nitric oxide / nitrite measurements by Griess assay in 3T3 L1 cells grown in different concentrations of exogenous L-Arg

**Fig. 7:**
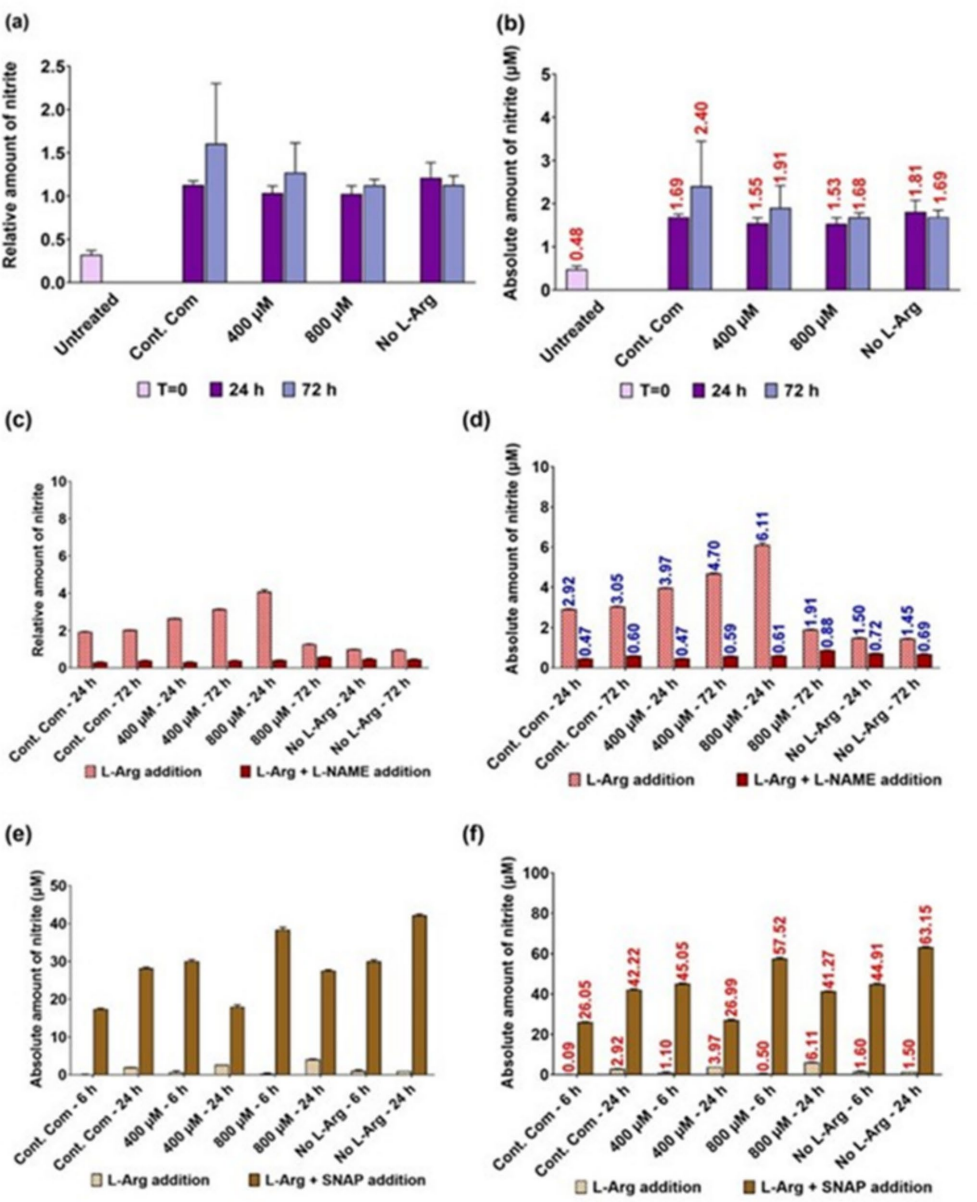
The effect of exogenous L-Arg concentration on nitrite production in 3T3 L1 cells. Cell culture supernatant was obtained from cultured 3T3 L1 cells grown in the presence of 0, 400 or 800 µM L-Arg and the controls complete DMEM addition for 24 or 72 h and untreated cell samples at T = 0 (relative (**a**) and absolute (**b**) values), cells grown in the presence of L-NAME (4 mM) in 0, 400 or 800 µM L-Arg and the control complete DMEM for 24 or 72 h (relative (**c**) and absolute (**d**) values) and cells grown in the presence of SNAP (100 µM) in 0, 400 or 800 µM L-Arg and the control complete DMEM for 6 or 24 h (relative (**e**) and absolute (**f**) values). Quantified nitrite was normalised to the nitrite amount presence in cultures with no L-Arg SILAC DMEM (0 µM L-Arg) at 24 h. Data points represent the mean ± SD. Error bars represent the standard deviation from the mean (n = 3)

The amount of nitrite present in the samples was normalised to the amount of nitrite present in the no L-Arg (0 µM) cell samples at 24 h and then the relative (Fig. 7a, c, and e) and the absolute (Fig. 7b, d, and f) amount of nitrite present in the cell samples are presented Figs. 7a–f. Interestingly, excess exogenous L-Arg addition increased (P < 0.0001) NO synthesis in 3T3 L1 cells (Fig. 7a and b). The amount of NO_2_^−^ presents in 400 (1.4-fold) and 800 µM (2.1-fold) L-Arg samples was high (P < 0.0001) at 24 h compared to the control complete DMEM cultured samples at the same time point in 3T3 L1 cells. At 72 h, the amount of nitrite presents in 400 µM L-Arg cultured samples was increased (NO_2_^−^; 4.70 µM, 1.54-fold), whereas in 800 µM was decreased (NO_2_^−^;1.91 µM, 0.62-fold) in comparison to the control complete DMEM cultured sample at 72 h (NO_2_^−^; 3.05 µM). However, the amount of nitrite presents in untreated samples at T = 0 (NO_2_^−^; 0.48 µM) and in 0 µM (24 h; NO_2_^−^; 1.45 µM and 72 h; NO_2_^−^; 1.50 µM) cultured samples was low regardless of the time point. It is noted that in regard to these overall findings, the amount of nitrite present in the samples was higher in L-Arg treated samples.

### Determination of nitric oxide / nitrite measurements by Griess assay in 3T3 L1 cells grown in different concentrations of exogenous L-Arg with L-NAME (4 mM) and SNAP (100 µM)

In Fig. 7c and d, the amount of NO decreased (P < 0.0001) in the samples cultured with L-NAME after 24 (the control + L-NAME; 0.16-fold, 400 µM + L-NAME; 0.12-fold, 800 µM + L-NAME; 0.10-fold and 0 µM + L-NAME; 0.48-fold) and 72 h (the control + L-NAME; 0.2-fold, 400 µM + L-NAME; 0.13-fold, 800 µM + L-NAME; 0.45-fold and 0 µM + L-NAME; 0.47-fold) compared to the samples cultured without L-NAME. Interestingly, a large drop (P < 0.0001) in the amount of nitrite was observed in the samples cultured in excess L-Arg 400 and 800 µM with L-NAME (0.47and 0.61 µM nitrite, respectively) in comparison to the samples cultured without L-NAME (400; 3.97 and 800 µM L-Arg; 6.11 µM nitrite) after 24 h.

Overall, upon addition of SNAP to 3T3 L1 cells cultured in L-Arg (0, 400 and 800 µM) there was a large increase (P < 0.0001) in the nitrite amount at 6 (the control + SNAP; 305.26-fold, 400 µM + SNAP; 40.66-fold; 800 µM + SNAP; 116.39-fold and 0 µM + SNAP; 28.02-fold) and 24 h (the control + SNAP; 14.46-fold, 400 µM + SNAP; 6.8-fold; 800 µM + SNAP; 6.75-fold and 0 µM + SNAP; 42.2-fold) compared to the cell samples cultured without NO donor at the same timepoints Fig. 7e and f.

### HPLC analysis of residue L-arginine, and L-ornithine and L-citrulline in the cell culture media of 3T3 L1 cells cultured in different initial L-Arg concentrations

**Fig. 8:**
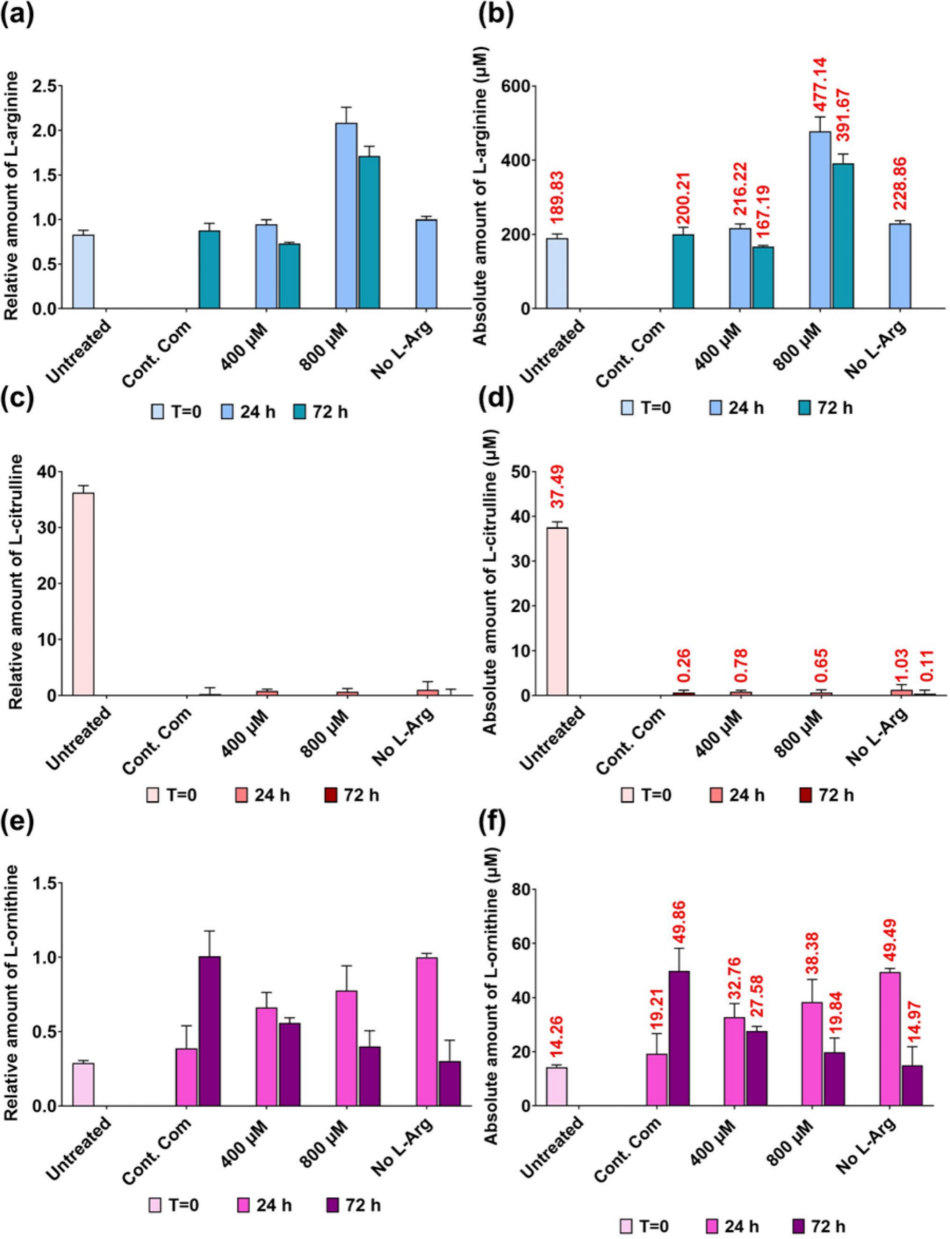
Residual serum L-Arg, L-Cit and L-Orn in 3T3 L1 cells with L-Arg. Relative and absolute residue amount of L-Arg (**a** and **b**), L-Cit (**c** and **d**) and L-Orn (**e** and **f**) analysed in plasma samples of 3T3 L1 cells cultured in different amount of L-Arg (0, 400 and 800 µM) and the control complete DMEM media after 24 and 72 h. Untreated cultures at T = 0. Data points represent the mean ± SD of each sample. Error bars represent the standard deviation from the mean (n = 3)

The amount of each target amino acid present in the samples was normalised to the amount of particular amino acid present in the no L-Arg (0 µM) cell samples at 24 h and then the relative and the absolute amount of target amino acids present in the cell samples are presented in the Fig. 8a–f.

L-Arg (Fig. 8a and b) was not detectable in samples cultured in the control complete DMEM at 24 h. The amount of L-Arg decreased with culture time in 400 and 800 µM L-Arg at 24 (287.27 µM and 243.14 µM, respectively) and 72 h (159.29 µM and 206.99 µM, respectively). At 72 h, L-Arg at 400 (0.74-fold) and 800 µM (0.96-fold) had decreased in comparison to the control at 72 h. However, at time point 72 h, samples cultured with no L-Arg had increased amount of L-Arg (1.09-fold) compared to the control at 72 h.

In the investigation of L-Cit (Fig. 8c and d), untreated samples at T = 0 had the highest amount of L-Cit (37.49 µM) among all samples. There was no detectable amount of L-Cit present in some cultured samples. The amount of L-Cit present in 800 µM L-Arg was higher (1.13-fold) compared to the control. At 24 h, there was no detectable amount of L-Cit present in the cultured samples except for samples cultured in no L-Arg (0.26 µM).

For the analysis of L-Orn (Figs. 8e and f), samples cultured in 0 µM L-Arg had the highest amount of L-Orn (49.86 µM) among all samples. The amount of L-Orn was increased in samples cultured in L-Arg (400 and 800 µM) and the control complete DMEM across the time points. However, L-Arg at 400 (24 h; 0.27-fold and 72 h 0.76-fold) and 800 µM (24 h; 0.76-fold and 72 h 0.52-fold) contained less L-Orn compared to the control samples cultured in complete DMEM at 24 and 72 h time points.

### HPLC analysis of residue L-arginine, and L-ornithine and L-citrulline in the cell culture media of 3T3 L1 cells cultured in different initial L-Arg with L-NAME (4 mM) and SNAP (100 µM)

**Fig. 9:**
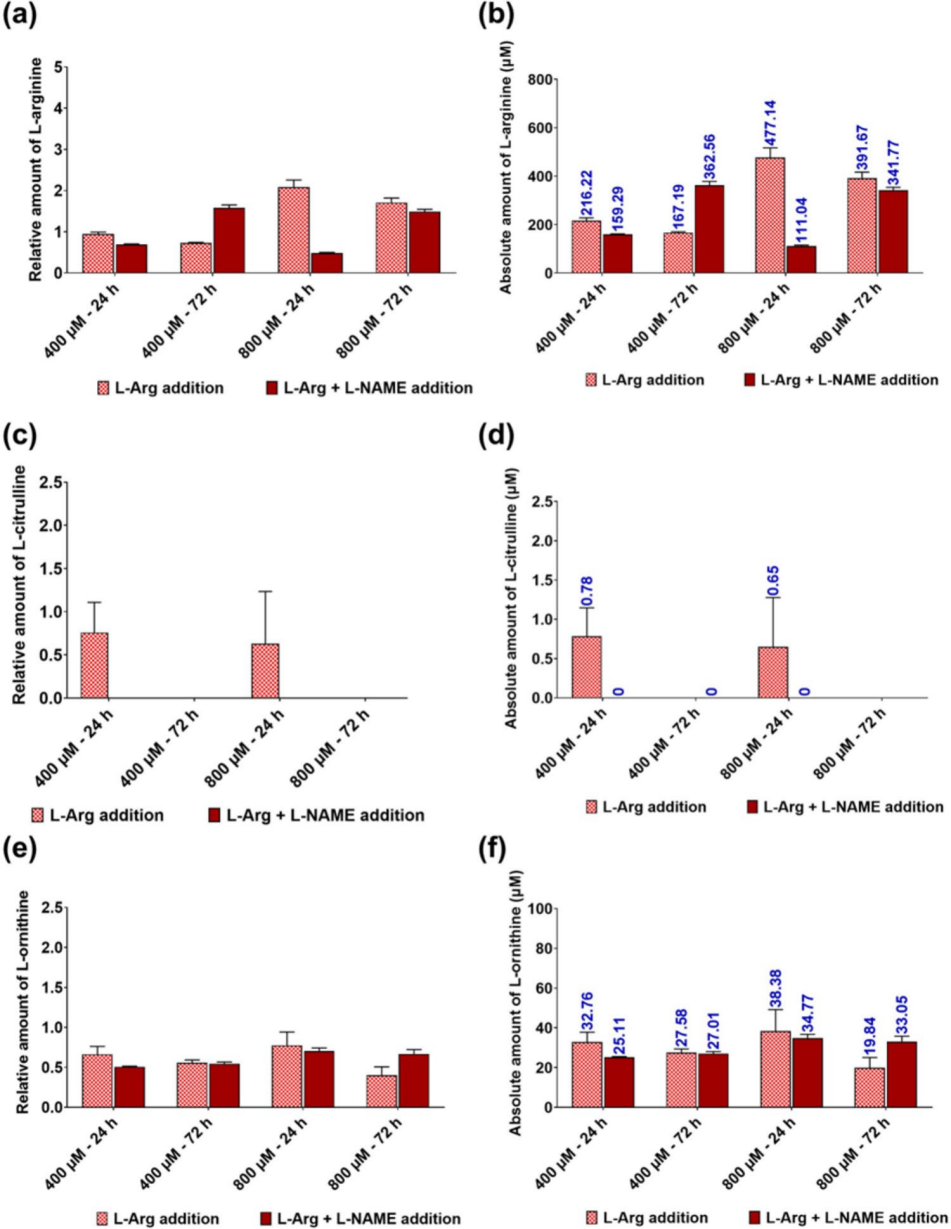
Residual serum L-Arg, L-Cit and L-Orn in 3T3 L1 cells with L-Arg and L-NAME (4 mM) for 24 and 72 h. Relative and absolute residue amount of L-Arg (**a** and **b**), L-Cit (**c** and** d**) and L-Orn (**e** and **f**) analysed in plasma samples of 3T3 L1 cells cultured with or without the presence of iNOS inhibitor; L-NAME (4 mM) in excess amount of L-Arg (400 and 800 µM) after 24 and 72 h. Data points represent the mean ± SD of each sample. Error bars represent the standard deviation from the mean (n = 3)

L-Arg concentrations in the cell culture supernatant samples (Fig. 9a, b) from the cells cultured in 400 and 800 µM L-Arg with L-NAME after 24 (0.74-fold and 0.24-fold, respectively) and 72 h (400 µM + L-NAME; 0.88-fold) was low, although in 400 µM L-Arg with L-NAME after 72 h (2.16-fold) this was increased compared to the samples cultured without L-NAME. L-Cit Fig. 9c, d was not detected (P < 0.001) in the samples cultured in L-Arg (400 and 800 µM) and L-NAME. When L-Orn concentrations were investigated (Fig. 9e, f) there was no significant changes (P = 0.8619) in concentration between samples cultured with and without L-NAME.

**Fig. 10:**
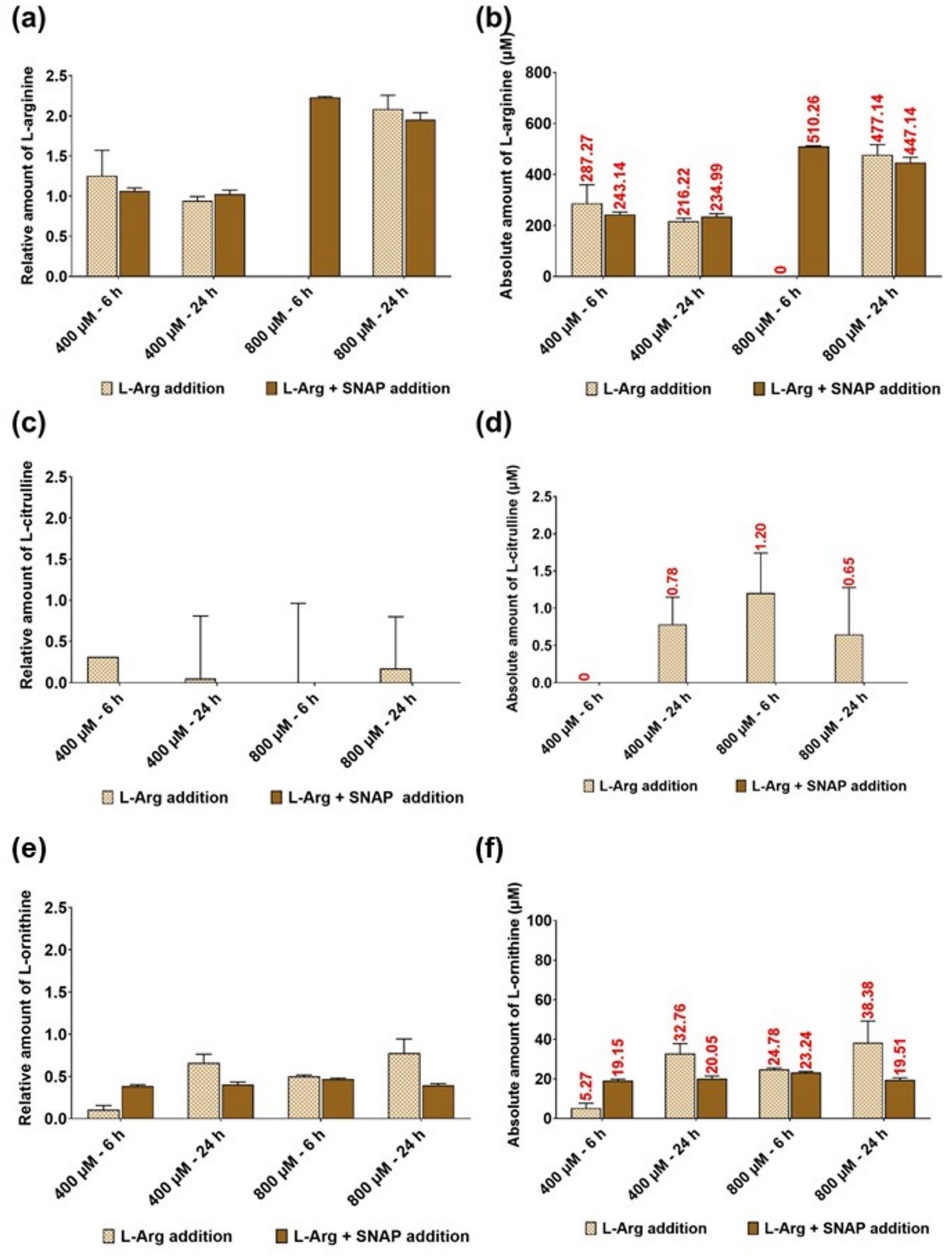
Residual serum L-Arg, L-Cit and L-Orn in 3T3 L1 cells with L-Arg and SNAP (100 µM) for 6 and 24 h. Relative and absolute residue amount of L-Arg (**a** and **b**), L-Cit (**c** and **d**) and L-Orn (**e** and **f**) analysed in plasma samples of 3T3 L1 cells cultured with or without the presence of external NO donor; SNAP (100 µM) in excess amount of L-Arg (400 and 800 µM) after 6 and 24 h. Data points represent the mean ± SD of each sample. Error bars represent the standard deviation from the mean (n = 3)

The cell culture supernatant concentration of L-Arg (Fig. 10a, b) in the samples cultured in L-Arg at 400 µM with SNAP was decreased (P < 0.0001) after 6 (400 µM + SNAP; 0.87-fold) but increased (P < 0.0001) after 24 h (1.1-fold). L-Cit was not detected with SNAP addition (Fig. 10c, d). The amount of L-Orn (Fig. 10e, f) was increased (P < 0.05) in the samples cultured with 400 µM L-Arg and SNAP at 6 h (400 µM + SNAP; 3.55-fold), whereas it was decreased (P < 0.05) after 24 h (400 µM + SNAP; 0.62-fold) in comparison to samples cultured without SNAP. Arginine at 800 µM had decreased (P < 0.05) L-Orn at 6 h (800 µM + SNAP; 0.94-fold) and 24 h (800 µM + SNAP; 0.5-fold).

## Discussion

Here the impact of excess exogenous L-Arg in the media of *in-vitro* cultured model cell lines and the subsequent impact on NO signalling and L-Arg metabolism has been investigated. Previous studies have reported that dietary arginine supplementation reduces fat mass in obese animal models (Fu et al. 2005) and humans with type-2 diabetes (Lucotti et al. 2006). The goal of this study was to investigate how excess L-Arg perturbed the L-Arg/NOS/NO metabolic pathways in *in-vitro* cultured insulin sensitive cells from the adipocytes (3T3 L1). The first part of the study was designed to evaluate the effect of L-Arg on metabolism of the cells and the second part was conducted to investigate the effect of modulators of NO synthesis (L-NAME and SNAP).

The absence of L-Arg resulted in a rapid drop in culture viability and decreased growth as expected, confirming the presence is required for growth of the cells. Addition of higher concentrations of L-Arg to the culture medium of 3T3 L1 cells does impact subsequent cell signalling in a concentration and time dependent manner. After 24 h, in 3T3 L1 cells cultures with additional 400 µM L-Arg concentration, the transcript levels of AMPK increased (Fig. 1c RE 3.26; P < 0.0001) compared to the control samples cultured in complete DMEM media. The results indicated that elevated L-Arg concentrations had an effect on AMPK expression, consistent with our BNL CL2 (mouse hepatocytes cells) findings (studied) and other findings reported that L-arginine increases the expression and activity of AMPK, thereby modulating lipid metabolism and energy balance (Tan 2012).

There was not a similar pattern for transcript (Fig. 1c) and protein (Fig. 4a) expression of AMPK in response to exogenous L-Arg concentrations. Changes in AMPK transcription before changes in protein synthesis are observed in many cultured conditions; this may reflect a time dependent link between the transcription and translation processes. This also reflects the multifaceted cell signalling pathways that converge and cross-talk with AMPK to coordinate responses to nutritional and hormonal signalling of the cells (Garcia and Shaw 2017). With regard to time dependent changes, it would be interesting in future studies to determine if the drop in transcript amounts in 400 µM cultures at 72 h resulted in a drop in protein amounts at a later time point for example. Further, in this study AMPK transcript expression was analysed by monitoring one of the catalytic subunits of AMPK isoforms; AMPKα1. However, in protein analysis, the primary antibody for anti-AMPK detects both isoforms of AMPKα (AMPKα1 and AMPKα2) but does not distinguish the isoforms present in the samples. This may also account for differences between the protein and transcript data.

Activated AMPK phosphorylated its downstream target ACC-1, the rate-controlling enzyme for the synthesis of malonyl-CoA, a precursor for de novo fatty acid synthesis (Viollet et al. 2006 and Su et al. 2014). When investigated phosphorylated AMPK protein, it is noted from the profiles that phosphorylated AMPK (Fig. 4c) and phosphorylated ACC-1 (Fig. 4d) levels in the control complete DMEM media were 2.07 and 1.48-fold higher respectively compared to the samples cultured in 400 µM L-Arg at time point 24 h. As a consequence, ACC-1 could be inactivated through its phosphorylation, allowing CPT-1 to act in its role in the transport of long-chain fatty acids to the mitochondria for oxidation (Viollet et al. 2006). In this scenario, in 3T3 L1 cells, perhaps unexpectedly, CPT-1A protein expression decreased in the control complete DMEM samples (studied) in comparison to the L-Arg cultures. However, CPT-1 mRNA expression did not show significant increases or decreases among cultures whilst the samples cultured in 400 and 800 µM L-Arg had a peak expression of CPT-1A protein after 24 h (studied). Interestingly, the effects of L-Arg on lipogenesis in adipocytes was reported in a study using 9-week-old male Zucker diabetic fatty rats, when rats were pair-fed with a specified diet and fed with drinking water containing arginine-HCl (1.51%) or alanine (2.55%, isonitrogenous control) for 10 weeks. The results indicated that the expression of major genes accountable for fatty acid oxidation in adipose tissue (e.g. AMPK and CPT-2) were increased by arginine supplementation (Fu et al. 2005). As discussed above, the additional results obtained from an experiment and analysis also show that, L-Arg increased the expression of CPT-1A indicating a potent effect of L-Arg on inhibiting adipose tissue fatty acid synthesis.

In 3T3 L1 cells, ACC-1 phosphorylation was low in the L-Arg treated samples compared to the control samples. Therefore, L-Arg treatment may increase free fatty acids availability in adipose tissue by reducing ACC-1 phosphorylation. Fatty acid synthesis is essentially high in adipocyte 3T3 L1 cells (Kim et al. 1996). Therefore, presumably L-Arg addition regulates fatty acid content in adipose tissue by decreasing ACC-1 phosphorylation, whereas, increasing expression of CPT-1 (studied) in L-Arg treated adipose tissues.

**Fig. 11:**
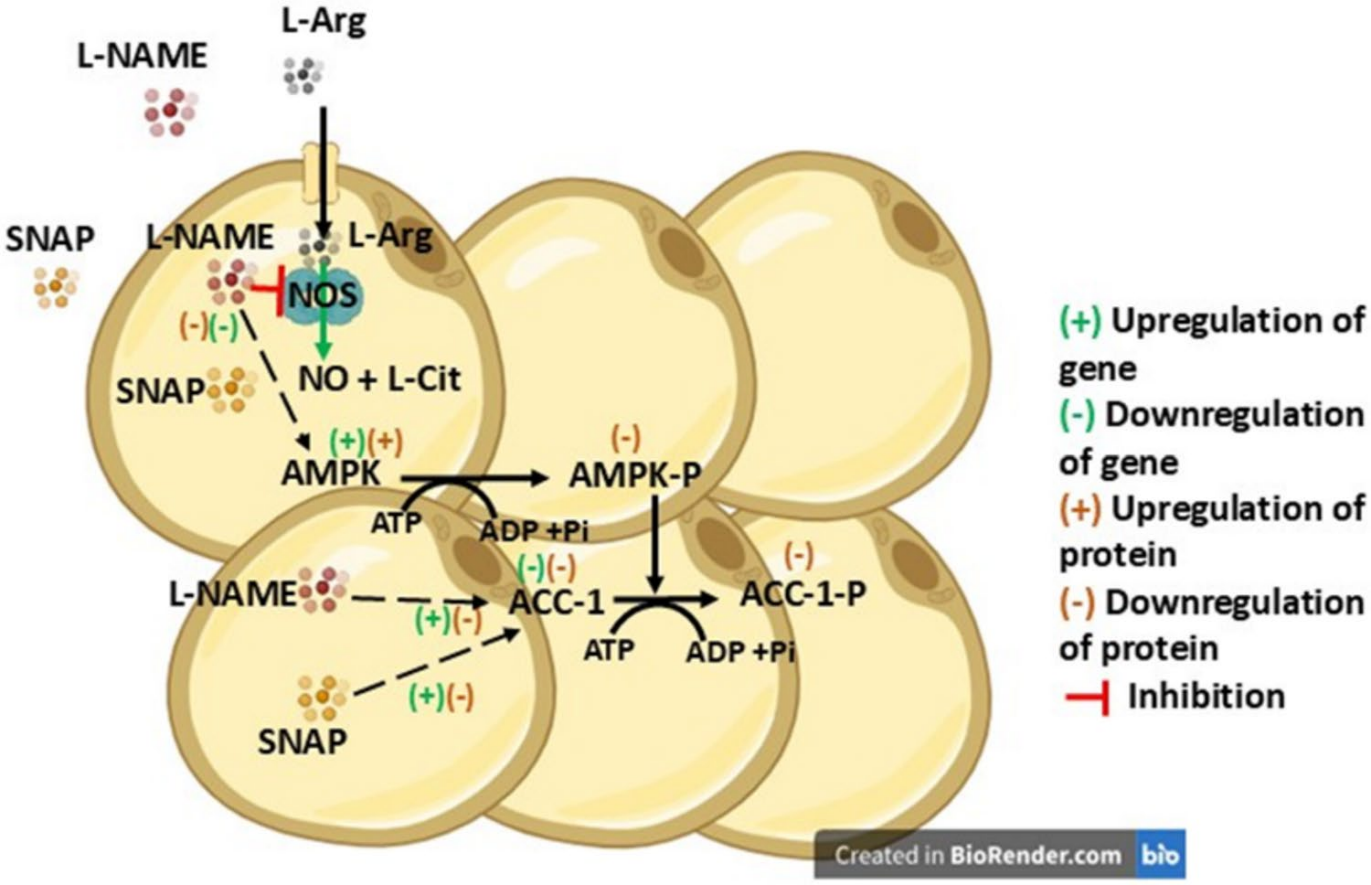
Putative AMPK and ACC-1 cell signalling pathway in L-Arg/NOS/NO (L-Arg at 800 µM) and regulation of this pathway by the modulators. The proposed mechanisms responsible for the beneficial effect of L-Arg/NOS/NO on AMPK and ACC-1 cell signalling pathways in mammalian adipose cells, 3T3 L1. The symbol (+) denotes an upregulation in gene expression or protein expression. The symbol (−) denotes a downregulation of gene expression or protein expression

In 3T3 L1 cells, inhibition of nitric oxide synthase enzyme activity by L-NAME, decreased the L-Arg-stimulated increases of AMPK gene (Fig. 2a) and protein (Fig. 5e) expression in 400 and 800 µM L-Arg at 24 and 72 h. The samples cultured in L-Arg deficient DMEM media and L-NAME showed high relative gene expression for AMPK (Fig. 2a) and ACC-1 (Fig. 2b) after 72 h but decreased protein levels (Fig. 5e and f). These results demonstrated that the L-Arg metabolic pathway and the AMPK/ACC-1 cell signalling pathway in the 3T3 L1 cells were regulated by the iNOS inhibitor L-NAME, which overall downregulated the transcript and protein levels of AMPK and ACC-1. In this study, external addition of NO via the NO donor, SNAP, upregulated ACC-1 transcript amounts (Fig. 2d). Noticeably, ACC-1 protein expression decreased after 24 h in the presence of SNAP (Fig. 6f). This likely reflects that the presence of NO either by metabolic pathway of L-Arg or addition of NO by the NO donor to the samples impacts transcriptional and translational expression of ACC-1. All these coordinated changes (all data included from the study) in gene and protein expression (summarized in Fig. 11) in one of the insulin-sensitive tissues; adipose tissue may partially provide a molecular mechanism for the fat mass deposition during obesity and how that would be overcome by L-Arg supplementation.

This study has a limitation, which is the small sample size (N = 3). As there are many variables (the control and 0, 400 and 800 µM L-Arg supplementations and different time points; T = 0, 24 and 72 h and T = 0, 24, 48 and 72 h), the least sample size; N = 3 was affordable due to some limiting factors such as the availability of funding and the time frame to complete my PhD. I declared that this study has some gaps that need to be fulfilled in future studies. As the introduction discusses, L-Arg is metabolized by two enzymes; NOS and arginase. This study focused on the NOS metabolic pathway and analysed the catabolic products of that pathway (the products of L-Arg/NOS; NO and L-Cit). It would be interesting to conduct a study in the future to analyse the L-Arg metabolism by arginase and its catabolic products (L-Arg/arginase; L-Ornithine and urea). Further, this study focused on the effect of L-Arg on AMPK/ACC-1 expressions, which have major roles in lipid metabolism. NO may regulate AMPK expression directly or indirectly. It would be interesting to do follow-up studies to examine the NO regulation of AMPK or the effect of AMPK inhibition on NO production along with the L-Arg addition. *In-vitro* studies such as those described here are important in that they allow laboratory-based studies in cell culture systems. Here it is shown that excess exogenous L-Arg (400 and 800 µM) impacts metabolism in mammalian adipose tissue. In the future, an *in-vivo* study to determine if these concentrations are safe and effective in intact organisms, when the animal model supplemented with different concentrations of L-Arg (400 and 800 µM) and the control L-alanine (as a negative control amino acid) and/or saline water would need to be undertaken to confirm the in vitro results transfer to a whole animal. From previous reports, in vivo studies have administered L-Arg at various doses to animals and humans, resulting in a range of plasma concentrations. Achieving the high concentrations used in in vitro systems may be challenging due to physiological limitations, but certain administration methods can elevate plasma levels significantly. There are two methods for L-Arg administration; one method is intravenous administration and the other is oral administration. For the former method, in humans, intravenous infusion of a 30 g (approximately 430 mg/kg for a 70 kg individual) L-Arg hydrochloride dissolved in 150 mL physiological saline or placebo (150 mL physiological saline) over 30 min increased plasma L-Arg concentrations from a baseline of 71 ± 4 μmol/L to a peak of 6,223 ± 407 μmol/L (Bode-Böger et al. 1998). Another study reported that for intravenous dose, 300 mL of a 10% L-Arg hydrochloride solution (the same 30 g) to humans raised plasma levels from 15.1 ± 2.6 μg/mL to 1,390 ± 596 μg/mL (Tangphao et al. 1999). Regarding the later administration method, a single oral dose of 6 g L-Arg or placebo (12 capsules of 0.5 g of L-Arg hydrochloride or lactose) to humans resulted in peak plasma concentrations of approximately 310 ± 152 μmol/L (Bode-Böger et al. 1998). Another study revealed that oral administration of 100 mL of a 10% L-Arg solution (10 g L-Arg) to humans led to peak plasma levels of 50.0 ± 1 3.4 μg/mL after 1 h administration (Tangphao et al. 1999). When considered L-Arg administration to animals, oral doses of 500 mg/kg/day for 4 weeks and 1000 mg/kg/day over 16 weeks to rats were studied. The higher dose did not significantly alter plasma L-Arg (194 ± 10 µM) levels compared to the control (220 ± 22 µM) (Kim et al. 2023). Oral administration of 0.2 to 0.8 g/kg/day twice daily between 0 and 14 days of age of piglets increased plasma L-Arg concentrations by 34 -195%, depending on the dose (Long et al. 2025).

Achievability of in vitro L-Arg concentrations to in vivo, in this in vitro study, L-Arg concentrations in the micromolar range (400–800 µM) were used. The highest plasma concentrations achieved in human studies via intravenous infusion (approximately 430 mg/kg) was 6, 223 µM but this value is transient and decline rapidly due to metabolism and renal clearance (Bode-Böger et al. 1998). Sustaining such high plasma levels over extended periods in vivo is challenging. Continuous intravenous infusion could maintain elevated levels, but this method is invasive and may not be practical for long-term studies. Oral administration results in lower peak concentrations and is subject to first-pass metabolism, reducing bioavailability (Stielow et al. 2023). Therefore, while short-term elevation of plasma L-Arg to concentrations used in vitro is possible through high-dose intravenous infusion, maintaining these levels over longer durations in vivo is limited by physiological factors. Alternative strategies, such as using L-citrulline supplementation to increase endogenous L-Arg levels, have been explored but also face limitations in achieving and sustaining high plasma concentrations (Rashid et al. 2020). Achieving and maintaining in vitro-like concentrations of L-Arg in vivo is constrained by metabolic and physiological processes, and while transient elevation is feasible, sustained high levels pose significant challenges.

## Supplementary Information

Below is the link to the electronic supplementary material.

Supplementary file1 (DOCX 42 KB)

## Data Availability

No datasets were generated or analysed during the current study.

## Abbreviations

3T3 L1: Mouse adipocytes spontaneously immortalized cell line
ACC: Acetyl-CoA carboxylase
AMPK: 5′-adenosine monophosphate-activated protein kinase
HPLC: High-performance liquid chromatography
L-Arg: L-arginine
L-Cit: L-citrulline
L-NAME: N^G^-nitro-L-Arg methylester
L-Orn: L-ornithine
NOS: Nitric oxide synthase
NO: Nitric oxide
NO_2_^−^: Nitrite
SILAC DMEM: Stable isotope labeling with amino acids in cell culture DMEM
SNAP: S-nitroso-N-acetyl-DL-penicillamine
