# Supplementary material for: The effect of L-Arg supplementation on L-Arg/NO metabolic and AMPK/ACC-1 signalling pathways in adipose cells (3T3 L1)
Source: Amino Acids. 2025 Aug 5;57:39. doi: 10.1007/s00726-025-03467-0 (PMC12325452; doi:10.1007/s00726-025-03467-0)
Supplement: Supplementary file 1 [file 726_2025_3467_MOESM1_ESM.docx]

**Supplementary Materials**

The effect of L-arginine supplementation on L-Arg/NO metabolic and AMPK/ACC-1 signalling pathways in adipose cells (3T3 L1), Amino Acids, Dr. Saranya Prashath, Centre for Genomic and Child Health, Queen Mary University of London, UK, s.prashath@qmul.ac.uk.

**Table. S1** Primers used for real-time qPCR analysis of the target genes.

| Target | Gene | Sequence (5'-3') |
| --- | --- | --- |
| ACC-1 | *Acaca* | Forward: GGGTCAAGTCCTTCCTGCTC |
|  |  | Reverse: TTCCACACACGAGCCATTCA |
| AMPK | *Prkaa1* | Forward: GGATCCATCAGCAACTATCG |
|  |  | Reverse: TCGACTCCTCCCCTGTCGAC |
| β-actin | *Actb* | Forward: AGCTGAGAGGGAAATTGTGCG |
|  |  | Reverse: GCAACGGAAACGCTCATT |

**Table. S2** List of antibodies used and sourced from different companies for Western blotting.

| Target protein | Type | Dilution | Source company | Catalog No |
| --- | --- | --- | --- | --- |
| Anti-ACC-1 | Primary | 1:1000 | GeneTex | GTX132081 |
| Anti-ACC-1-P (Ser79) | Primary | 1:1000 | GeneTex | GTX133974 |
| Anti-AMPK | Primary | 1:1000 | Cell Signaling | 2532 |
| Anti-AMPK-P (Thr172) | Primary | 1:500 | Cell Signaling | 2535 |
| Anti-β-actin | Primary | 1:1000 | Sigma | A5441 |
| Anti-mouse IgG-Peroxidase | Secondary | 1:5000 | Sigma | A4416 |
| Anti-rabbit IgG-Peroxidase | Secondary | 1:5000 | Sigma | A6154 |

**Table. S3** HPLC gradient program for separation of L-Arg, L-Cit and L-Orn at flow rate 1.1 mL/min.

| **Mobile**  **Phase (%)** | **Time (min)** | | | | | | | | | | | |
| --- | --- | --- | --- | --- | --- | --- | --- | --- | --- | --- | --- | --- |
|  | 0 | 15 | 20 | 24 | 26 | 34 | 38 | 40 | 42 | 42.1 | 49 |  |
| **A** | 86 | 86 | 70 | 65 | 53 | 50 | 30 | 0 | 0 | 86 | 86 |  |
| **B** | 14 | 14 | 30 | 35 | 47 | 50 | 70 | 100 | 100 | 14 | 14 |  |

The effect of L-arginine supplementation on L-Arg/NO metabolic and AMPK/ACC-1 signalling pathways in adipose cells (3T3 L1), Amino Acids, Dr. Saranya Prashath, Centre for Genomic and Child Health, Queen Mary University of London, UK, [s.prashath@qmul.ac.uk](mailto:s.prashath@qmul.ac.uk).

**Statistical Analysis Tables**

**Table. 1.1**

| Source of Variation | P value | P value summary | Significant? |
| --- | --- | --- | --- |
| Interaction | <0.0001 | **** | Yes |
| Time point | <0.0001 | **** | Yes |
| L-Arg +/- and Control (Com) | <0.0001 | **** | Yes |

**Table. 1.2**

| **Samples comparison** | **Variables: Cell fitness parameters** | | | | | | | |
| --- | --- | --- | --- | --- | --- | --- | --- | --- |
|  | **Cell growth** | | | | **Culture viability** | | | |
|  | **T=24 h** | **T=48 h** | **T=72 h** | **T=120 h** | **T=24 h** | **T=48 h** | **T=72 h** | **T=120 h** |
| **Cont. Com vs. 400 µM** | * | **** | ns | ** | ns | ns | ns | ** |
| **Cont. Com vs. 800 µM** | ns | *** | ns | ns | ns | ns | * | *** |
| **Cont. Com vs. No L-Arg** | **** | **** | **** | **** | ns | *** | *** | **** |

**Tables. 1.1 and 1.2** Cell growth/viable cell number and culture viability profiles of 3T3 L1 cells with different concentrations of L-arginine (0, 400 and 800 µM) and the control complete DMEM media at 24, 48, 72 and 120 h. The tables summarise two-way ANOVA followed by a Tukey multiple comparison test using GraphPad Prism 9.4.1. The stars flag the levels of significance; ns = P > 0.05, * = P ≤ 0.05, ** = P ≤ 0.01, *** = P ≤ 0.001 and **** = P ≤ 0.0001.

**Table. 2.1**

|  | AMPK | | | ACC-1 | |
| --- | --- | --- | --- | --- | --- |
| Source of Variation | | **P value** | **Significant?** | **P value** | **Significant?** |
| Interaction | | **** | Yes | **** | Yes |
| Time point | | **** | Yes | **** | Yes |
| L-Arg +/- and control (Com) | | **** | Yes | **** | Yes |

**Table. 2.2**

| **Samples comparison** | **Variables: Gene expression** | | | | | |
| --- | --- | --- | --- | --- | --- | --- |
|  | **AMPK** | | | **ACC-1** | | |
|  | **T=0** | **T=24 h** | **T=72 h** | **T=0** | **T=24 h** | **T=72 h** |
| **Cont. Com vs. untreated** | ns | ns | ns | **** | **** | **** |
| **Cont. Com vs. 400 µM** | ns | **** | ns | ns | ns | **** |
| **Cont. Com vs. 800 µM** | ns | *** | ns | ns | ns | **** |
| **Cont. Com vs. No L-Arg** | ns | ns | ns | ns | ** | **** |

**Tables. 2.1 and 2.2** Relative mRNA transcript expression (∆∆Ct) of AMPK and ACC-1 in 3T3 L1 cells cultured in medium of different L-arginine concentrations (0, 400 and 800 µM) and control complete DMEM media 24 and 72 h after addition. Untreated cultures at T=0. Tables summarise two-way ANOVA followed by a Tukey multiple comparison test using GraphPad Prism 9.4.1. The stars indicate the levels of significance; ns = P > 0.05, * = P ≤ 0.05, ** = P ≤ 0.01, *** = P ≤ 0.001 and **** = P ≤ 0.0001.

**Table. 3.1**

|  | AMPK | | | ACC-1 | |
| --- | --- | --- | --- | --- | --- |
| Source of Variation | | **P value** | **Significant?** | **P value** | **Significant?** |
| Interaction | | **** | Yes | **** | Yes |
| Samples, conditions and time points | | **** | Yes | **** | Yes |
| L-Arg +/- SNAP | | ns | No | **** | Yes |

**Table. 3.2**

| **Samples comparison** |  |  |  |  |
| --- | --- | --- | --- | --- |
|  | **L-Arg addition and L-Arg + L-NAME addition** | | **L-Arg addition and L-Arg + SNAP addition** | |
|  | **AMPK** | **ACC-1** | **AMPK** | **ACC-1** |
| **Cont. Com - 24 h** | ns | **** | ns | * |
| **Cont. Com - 72 h** | ns | **** | * | ns |
| **400 µM - 24 h** | **** | **** | *** | **** |
| **400 µM - 72 h** | ns | **** | *** | ** |
| **800 µM - 24 h** | *** | ns | * | **** |
| **800 µM - 72 h** | ns | **** | ns | *** |
| **No L-Arg - 24 h** | ns | ns | ns | **** |
| **No L-Arg - 24 h** | ns | **** | ns | ns |

**Tables. 3.1 and 3.2** Relative mRNA transcript expression (∆∆Ct) of AMPK and ACC-1 in 3T3 L1 cells cultured in medium of different L-arginine concentrations (0, 400 and 800 µM) and control complete DMEM media with nitric oxide synthase inhibitor; L-NAME (4 mM) for 24 and 72 h after addition and the NO donor SNAP (100 µM) for 6 and 24 h. The tables summarise two-way ANOVA followed by a Tukey multiple comparison test using GraphPad Prism 9.4.1. The stars indicate the levels of significance; ns = P > 0.05, * = P ≤ 0.05, ** = P ≤ 0.01, *** = P ≤ 0.001 and **** = P ≤ 0.0001.

**Table. 4.1**

|  | AMPK | | AMPK-P | |
| --- | --- | --- | --- | --- |
| Source of Variation | **P value** | **Significant?** | **P value** | **Significant?** |
| Interaction | **** | Yes | **** | Yes |
| Time | **** | Yes | **** | Yes |
| L-Arg +/- and control (Com) | **** | Yes | **** | Yes |

**Table. 4.2**

|  | ACC-1 | | ACC-1-P | |
| --- | --- | --- | --- | --- |
| Source of Variation | **P value** | **Significant?** | **P value** | **Significant?** |
| Interaction | **** | Yes | **** | Yes |
| Time | **** | Yes | **** | Yes |
| L-Arg +/- and control (Com) | **** | Yes | **** | Yes |

**Table. 4.3**

| **Samples comparison** | **Variables: Protein expression** | | | | | | | | | | | |
| --- | --- | --- | --- | --- | --- | --- | --- | --- | --- | --- | --- | --- |
|  | **AMPK** | | | **AMPK-P** | | | **ACC-1** | | | **ACC-1-P** | | |
|  | **T=0** | **T=24 h** | **T=72 h** | **T=0** | **T=24 h** | **T=72 h** | **T=0** | **T=24 h** | **T=72 h** | **T=0** | **T=24 h** | **T=72 h** |
| **Cont. Com vs. untreated** | **** | **** | **** | **** | **** | **** | **** | **** | **** | ns | **** | **** |
| **Cont. Com vs. 400 µM** | ns | **** | **** | ns | **** | **** | ns | ns | * | ns | **** | * |
| **Cont. Com vs. 800 µM** | ns | ns | **** | ns | **** | **** | ns | ns | ** | ns | **** | **** |
| **Cont. Com vs. No L-Arg** | ns | **** | ns | ns | **** | **** | ns | **** | **** | ns | **** | **** |

**Tables. 4.1, 4.2 and 4.3** Relative protein amounts for total AMPKα and ACC-1 and phosphorylated AMPKα at Thr172 (AMPKα-P) and ACC-1 at Ser79 (ACC-1-P) in 3T3 L1 cells. The tables summarise two-way ANOVA followed by a Tukey multiple comparison test using GraphPad Prism 9.4.1. The stars indicate the levels of significance; ns = P > 0.05, * = P ≤ 0.05, ** = P ≤ 0.01, *** = P ≤ 0.001 and **** = P ≤ 0.0001.

**Table. 5.1**

| **Source of Variation** | **AMPK** | | | **ACC-1** | | |
| --- | --- | --- | --- | --- | --- | --- |
|  | **P value** | **P value summary** | **Significant?** | **P value** | **P value summary** | **Significant?** |
| **Interaction** | <0.0001 | **** | Yes | <0.0001 | **** | Yes |
| **Samples, conditions and time points** | <0.0001 | **** | Yes | <0.0001 | **** | Yes |
| **L-Arg +/- L-NAME** | <0.0001 | **** | Yes | <0.0001 | **** | Yes |

**Table. 5.2**

| **Source of Variation** | **AMPK** | | | **ACC-1** | | |
| --- | --- | --- | --- | --- | --- | --- |
|  | **P value** | **P value summary** | **Significant?** | **P value** | **P value summary** | **Significant?** |
| **Interaction** | <0.0001 | **** | Yes | <0.0001 | **** | Yes |
| **Samples, conditions and time points** | 0.0301 | * | Yes | <0.0001 | **** | Yes |
| **L-Arg +/- SNAP** | 0.0908 | ns | No | <0.0001 | **** | Yes |

**Table. 5.3**

| **Samples comparison** |  |  |  |  |
| --- | --- | --- | --- | --- |
|  | **L-Arg addition and L-Arg + L-NAME addition** | | **L-Arg addition and L-Arg + SNAP addition** | |
|  | **AMPK** | **ACC-1** | **AMPK** | **ACC-1** |
| **Cont. Com - 24 h** | **** | **** | *** | **** |
| **Cont. Com - 72 h** | ns | **** |  |  |
| **400 µM - 24 h** | * | **** | ns | **** |
| **400 µM - 72 h** | ns | **** |  |  |
| **800 µM - 24 h** | **** | **** | ns | **** |
| **800 µM - 72 h** | **** | **** |  |  |
| **No L-Arg - 24 h** | **** | **** | ** | **** |
| **No L-Arg - 72 h** | **** | ns |  |  |

**Tables. 5.1 - 5.3** Relative mRNA transcript expression (∆∆Ct) of AMPK and ACC-1 in 3T3 L1 cells cultured in medium of different L-arginine concentrations (0, 400 and 800 µM) and control complete DMEM media with addition of either L-NAME (4mM) or SNAP (100 µM) in 3T3 L1 cells across the time points either 24 and 72 h or 6 and 24 h. The tables summarise two-way ANOVA followed by a Bonferroni's multiple comparisons test using GraphPad Prism 9.4.1. The stars indicate the levels of significance; ns = P > 0.05, * = P ≤ 0.05, ** = P ≤ 0.01, *** = P ≤ 0.001 and **** = P ≤ 0.0001.

**Table. 6.1**

| Source of Variation | P value | P value summary | Significant? |
| --- | --- | --- | --- |
| Interaction | <0.0001 | **** | Yes |
| Time | <0.0001 | **** | Yes |
| L-Arg +/- and Control (Com) | 0.0002 | **** | Yes |

**Table. 6.2**

| Source of Variation | P value | | P value summary | Significant? |
| --- | --- | --- | --- | --- |
| Interaction | | <0.0001 | **** | Yes |
| Time | | <0.0001 | **** | Yes |
| L-Arg +/- and Control (Com) | | <0.0001 | **** | Yes |

**Table. 6.3**

| Source of Variation | P value | | P value summary | Significant? |
| --- | --- | --- | --- | --- |
| Interaction | | <0.0001 | **** | Yes |
| Time | | <0.0001 | **** | Yes |
| L-Arg +/- and Control (Com) | | <0.0001 | **** | Yes |

**Table. 6.4**

| Source of Variation | P value | | P value summary | Significant? |
| --- | --- | --- | --- | --- |
| Interaction | | <0.0001 | **** | Yes |
| Time | | <0.0001 | **** | Yes |
| L-Arg +/- and Control (Com) | | <0.0001 | **** | Yes |

**Table. 6.5**

| **Samples comparison** | **Nitrite** | | | **Serum L-Arg** | | | **Serum L-Cit** | | | **Serum L-Orn** | | |
| --- | --- | --- | --- | --- | --- | --- | --- | --- | --- | --- | --- | --- |
|  | **T=0** | **T=24 h** | **T=72 h** | **T=0** | **T=24 h** | **T=72 h** | **T=0** | **T=24 h** | **T=72 h** | **T=0** | **T=24 h** | **T=72 h** |
| **Cont. Com vs. untreated** | ns | **** | **** | **** | ns | **** | **** | ns | ns | ** | *** | **** |
| **Cont. Com vs. 400 µM** | ns | ns | ns | ns | **** | * | ns | ns | ns | ns | ** | **** |
| **Cont. Com vs. 800 µM** | ns | ns | ns | ns | **** | **** | ns | ns | ns | ns | *** | **** |
| **Cont. Com vs. No L-Arg** | ns | ns | ns | ns | **** | **** | ns | ns | ns | ns | **** | **** |

**Tables. 6.1 - 6.5** The effect of exogenous L-arginine concentration on nitrite production and quantification of residual serum L-Arg, L-Cit and L-Orn obtained from cultured 3T3 L1 cells grown in the presence of 0, 400 or 800 µM L-arginine for T=0, 24 or 72 h. Tables summarise two-way ANOVA followed by a Tukey multiple comparison test using GraphPad Prism 9.4.1. The stars indicate the levels of significance; ns = P > 0.05, * = P ≤ 0.05, ** = P ≤ 0.01, *** = P ≤ 0.001 and **** = P ≤ 0.0001.

**Table. 7.1**

| **Source of Variation** | **L-Arg + L-NAME** | | | **L-Arg + SNAP** | | |
| --- | --- | --- | --- | --- | --- | --- |
|  | **P value** | **P value summary** | **Significant?** | **P value** | **P value summary** | **Significant?** |
| **Interaction** | <0.0001 | **** | Yes | <0.0001 | **** | Yes |
| **Samples, conditions and time points** | <0.0001 | **** | Yes | <0.0001 | **** | Yes |
| **L-Arg +/- L-NAME/SNAP** | <0.0001 | **** | Yes | <0.0001 | **** | Yes |

**Table. 7.2**

| **Source of Variation** | **L-Arg + L-NAME** | | | **L-Arg + SNAP** | | |
| --- | --- | --- | --- | --- | --- | --- |
|  | **P value** | **P value summary** | **Significant?** | **P value** | **P value summary** | **Significant?** |
| **Interaction** | <0.0001 | **** | Yes | <0.0001 | **** | Yes |
| **Samples, conditions and time points** | <0.0001 | **** | Yes | <0.0001 | **** | Yes |
| **L-Arg +/- L-NAME/SNAP** | <0.0001 | **** | Yes | <0.0001 | **** | Yes |

**Table. 7.3**

| **Source of Variation** | **L-Arg + L-NAME** | | | **L-Arg + SNAP** | | |
| --- | --- | --- | --- | --- | --- | --- |
|  | **P value** | **P value summary** | **Significant?** | **P value** | **P value summary** | **Significant?** |
| **Interaction** | 0.003 | ** | Yes | 0.0045 | ** | Yes |
| **Samples, conditions and time points** | <0.0001 | **** | Yes | 0.0427 | * | Yes |
| **L-Arg +/- L-NAME/SNAP** | 0.0007 | *** | Yes | <0.0001 | **** | Yes |

**Table. 7.4**

| **Source of Variation** | **L-Arg + L-NAME** | | | **L-Arg + SNAP** | | |
| --- | --- | --- | --- | --- | --- | --- |
|  | **P value** | **P value summary** | **Significant?** | **P value** | **P value summary** | **Significant?** |
| **Interaction** | 0.0099 | ** | Yes | <0.0001 | **** | Yes |
| **Samples, conditions and time points** | 0.0084 | ** | Yes | <0.0001 | **** | Yes |
| **L-Arg +/- L-NAME/SNAP** | 0.8619 | ns | No | 0.0159 | * | Yes |

**Table. 7.5**

| **Samples comparison** | **L-Arg addition and L-Arg + L-NAME addition** | | |  |
| --- | --- | --- | --- | --- |
|  | **Amount of Nitrite** | **Amount of L-Arg** | **Amount of L-Cit** | **Amount of L-Cit** |
| **Cont. Com - 24 h** | **** |  |  |  |
| **Cont. Com - 72 h** | **** |  |  |  |
| **400 µM - 24 h** | **** | ** | * | ns |
| **400 µM - 72 h** | **** | **** | ns | ns |
| **800 µM - 24 h** | **** | **** | ns | ns |
| **800 µM - 72 h** | **** | * | ** | * |
| **No L-Arg - 24 h** | **** |  |  |  |
| **No L-Arg - 72 h** | **** |  |  |  |

**Table. 7.6**

| **Samples comparison** | **L-Arg addition and L-Arg + SNAP addition** | | |  |
| --- | --- | --- | --- | --- |
|  | **Amount of Nitrite** | **Amount of L-Arg** | **Amount of L-Cit** | **Amount of L-Orn** |
| **Cont. Com - 6 h** | **** |  |  |  |
| **Cont. Com - 24 h** | **** |  |  |  |
| **400 µM - 6 h** | **** | ns | ns | ** |
| **400 µM - 24 h** | **** | ns | *** | * |
| **800 µM - 6 h** | **** | **** | **** | ns |
| **800 µM - 24 h** | **** | ns | **** | *** |
| **No L-Arg - 6 h** | **** |  |  |  |
| **No L-Arg - 24 h** | **** |  |  |  |

**Tables. 7.1 – 7.6** The effect of exogenous L-arginine concentration on nitrite production and quantification of residual serum L-Arg, L-Cit and L-Orn obtained from cultured 3T3 L1 cells grown in the presence of 0, 400 or 800 µM L-arginine and the control complete DMEM media with addition of either L-NAME (4mM) or SNAP (100 µM) in 3T3 L1 cells across the time points either 24 and 72 h or 6 and 24 h. Tables summarise two-way ANOVA followed by a Bonferroni's multiple comparisons test using GraphPad Prism 9.4.1. The stars indicate the levels of significance; ns = P > 0.05, * = P ≤ 0.05, ** = P ≤ 0.01, *** = P ≤ 0.001 and **** = P ≤ 0.0001.
